# Parental education and children’s depression, anxiety, and ADHD traits, a within-family study in MoBa

**DOI:** 10.1038/s41539-024-00260-8

**Published:** 2024-07-18

**Authors:** Amanda M. Hughes, Fartein Ask Torvik, Elsje van Bergen, Laurie J. Hannigan, Elizabeth C. Corfield, Ole A. Andreassen, Eivind Ystrom, Helga Ask, George Davey Smith, Neil M. Davies, Alexandra Havdahl

**Affiliations:** 1 MRC Integrative Epidemiology Unit, Population Health Sciences, University of Bristol, Bristol, UK; 2 Centre for Fertility and Health, Norwegian Institute of Public Health, Oslo, Norway; 3 Promenta Research Centre, Department of Psychology, University of Oslo, Oslo, Norway; 4 Department of Biological Psychology, Vrije Universiteit Amsterdam, Amsterdam, the Netherlands; 5 Nic Waals Institute, Lovisenberg Diaconal Hospital, Oslo, Norway; 6 Population Health Sciences, Bristol Medical School, University of Bristol, Bristol, UK; 7 PsychGen Centre for Genetic Epidemiology and Mental Health, Norwegian Institute of Public Health, Oslo, Norway; 8 NORMENT Centre, Institute of Clinical Medicine, University of Oslo and Division of Mental Health and Addiction, Oslo University Hospital, 0407 Oslo, Norway; 9 KG Jebsen Centre for Neurodevelopmental disorders, University of Oslo, Oslo, Norway; 10 Division of Psychiatry, University College London, London, UK; 11 Department of Statistical Science, University College London, London, UK; 12 K.G. Jebsen Center for Genetic Epidemiology, Department of Public Health and Nursing, Norwegian University of Science and Technology, Trondheim, Norway

**Keywords:** Education, Human behaviour

## Abstract

Children born to parents with fewer years of education are more likely to have depression, anxiety, and attention-deficit hyperactivity disorder (ADHD), but it is unclear to what extent these associations are causal. We estimated the effect of parents’ educational attainment on children’s depressive, anxiety, and ADHD traits at age 8 years, in a sample of 40,879 Norwegian children born in 1998–2009 and their parents. We used within-family Mendelian randomization, which employs genetic variants as instrumental variables, and controlled for direct genetic effects by adjusting for children’s polygenic indexes. We found little evidence that mothers’ or fathers’ educational attainment independently affected children’s depressive, anxiety, or ADHD traits. However, children’s own polygenic scores for educational attainment were independently and negatively associated with these traits. Results suggest that differences in these traits according to parents’ education may reflect direct genetic effects more than genetic nurture. Consequences of social disadvantage for children’s mental health may however be more visible in samples with more socioeconomic variation, or contexts with larger socioeconomic disparities than present-day Norway. Further research is required in populations with more educational and economic inequality and in other age groups.

## Introduction

Children from socioeconomically disadvantaged families are more likely to have emotional and behavioural difficulties than their more advantaged peers^1,2^. Social inequalities in childhood exist for traits and diagnoses of emotional and behavioural conditions, including depression, anxiety^3^, and attention-deficit hyperactivity disorder (ADHD)^4,5^. Social inequalities have been reported according to parental education^6–8^, household income^3,4,9^, and other socioeconomic measures including housing tenure^10^. Findings like these suggest that intervening on children’s socioeconomic circumstances could lead to improvements in child emotional and behavioural functioning, but knowing which aspect of the environment to target is not straightforward. Firstly, socioeconomic factors like parental education and family income tend to be highly correlated, making it challenging to separate their individual effects. Secondly, associations with socioeconomic conditions can be biased by other aspects of a child’s environment. For instance, a parent’s depression may impact a family’s socioeconomic circumstances, whilst affecting their children’s emotional and behavioural difficulties via separate pathways (such as parenting behaviours)^11^. Thirdly, as well as shaping children’s environments, parents transmit 50% of their genomes to their children. Educational attainment is influenced by genetic factors: twin studies suggest approximately 43% of variation in educational attainment may reflect genetic differences^12^, and genome-wide association studies (GWAS) have identified common genetic variants explaining 12–16% of the variation in adult educational attainment^13^. This means that genetic inheritance (transmission of genetic factors from parents to children)^14^ may also contribute to any link between parents’ education and children’s outcomes.

To disentangle parental influences which operate through children’s environments from direct genetic effects, research has increasingly employed family-based approaches, such as the children-of-twins method and its extensions^14^. Children-of-twins studies rely on the fact that children of monozygotic twins are equally related to their parent and to their parent’s co-twin to isolate the impact of environmental factors, such as parenting behaviours and parental mental health^14,15^. A recent children-of-twins study suggested that maternal and paternal education influence children’s ADHD traits, but not their depressive traits, once direct genetic effects and shared environmental risk factors were accounted for^16^. When environmental exposures are influenced by the parents’ own genotype, their impact on children’s outcomes can be considered a case of genetic nurture, or dynastic effects: an indirect effect of parents’ or other relatives’ genotype on children’s traits via environmental pathways. Other studies have used molecular genetic data on multiple members of the same family to investigate genetic nurture effects after accounting for direct genetic transmission. This suggests parental influence on depressive^17^ and ADHD traits^18^, but not anxiety traits^17^, but these studies did not distinguish the role of specific parental phenotypes or behaviours. Other work examining genetic nurture effects report little evidence that parental educational attainment influences children’s ADHD traits^19,20^ but suggest children’s own genetic liability for educational attainment does^20^.

**Fig. 1:**
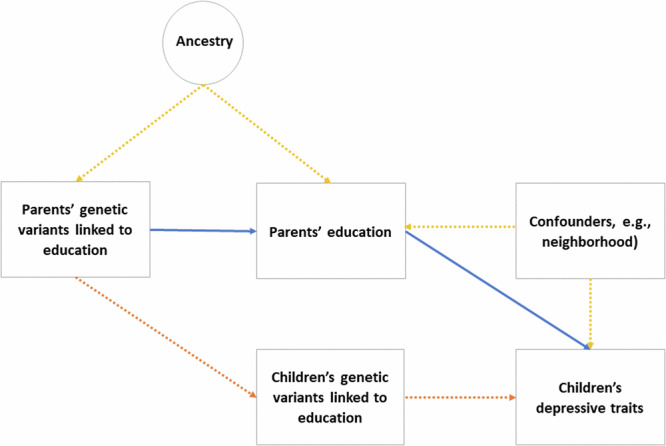
Mendelian randomization applied to an intergenerational relationship. Blue lines: genetic nurture. Orange lines: direct genetic transmission. Yellow lines: confounding by environmental factors or ancestry. Dotted lines: pathways accounted for by use of instrumental variables or adjustment. In Mendelian randomization, genetic variants associated with an exposure are used as instrumental variables for the exposure. Given certain assumptions, this avoids confounding by factors which influence both the exposure (here: parental educational attainment) and the outcome (here: children’s depressive traits). With intergenerational relationships, estimates can still be biased if genetic variants which influence the exposure in parents also influence the outcome when inherited by children. Within-family Mendelian randomization avoids this source of bias by adjusting for the child’s genotype. Ancestry can also confound associations of genetic variants with outcomes, so is typically adjusted for via principal components^24^.

Another approach which can be used to estimate the consequences of educational attainment is Mendelian randomization (MR), which uses genetic variants associated with an exposure as instrumental variables for that exposure^21^. A person’s germline genetic code is determined at conception and does not change in response to health or environmental conditions. Consequently, associations of outcomes with genetic variants are not affected by classical kinds of confounding or reverse causation. MR can be applied to study intergenerational relationships^22^, including the impact of parents’ educational attainment on their children’s emotional and behavioural difficulties. However, to separate the consequences of parents’ education from the impact of inherited variants in children, genetic data on large numbers of parents and children is required; to separate maternal from paternal effects requires genetic data on large numbers of mothers, fathers, and children (Fig. 1). Few studies have such data, which has limited the application of MR to intergenerational associations^23^.

We investigate the impact of one aspect of family socioeconomic position – parents’ educational attainment - on children’s depressive, anxiety, and ADHD traits. We use a genotyped sample of 40,879 mother-father-child trios from the Norwegian Mother, Father, and Child Cohort Study (MoBa). We apply a within-family Mendelian randomization (within-family MR) design to investigate the impact of maternal and paternal educational attainment on children’s depressive, anxiety, and ADHD traits at age 8 years. We instrument parental education using a polygenic index (PGI) comprising genetic variants previously linked to educational attainment, minimizing the impact of classical confounding and reverse causation. We account for direct genetic effects by adjusting for the same genetic variants in the children. By including the genotype of both parents, we estimate the conditional effects of both mothers’ and fathers’ educational attainment. Simultaneously, this approach provides an unconfounded estimate of the direct effect of these genetic variants on the children’s own depressive, anxiety, and ADHD traits.

Genetic studies designed to assess causation can be biased by horizontal pleiotropy: when genetic variants in a PGI influence the outcome via pathways which do not involve the exposure^21^. For instance, variants linked to parental education may also affect parental depression, plausibly affecting children’s traits independently of their parents’ education. In additional analyses, we therefore apply ‘two-sample’ Mendelian randomization methods, designed to assess horizontal pleiotropy by comparing associations of individual genetic variants with exposures and outcomes^24^.

## Results

### Descriptive characteristics

The Norwegian Mother, Father and Child Cohort Study (MoBa) is a population-based pregnancy cohort study conducted by the Norwegian Institute of Public Health^25^ which recruited from all over Norway from 1999 to 2008, attaining a participation rate of 41%. Previous comparison of the MoBa cohort with all women giving birth in Norway has shown that MoBa participants differed from the general population on demographic and health-related factors: mothers who were younger or living alone were underrepresented, as were smokers, and women who had had more previous pregnancies. Women who used multivitamins were overrepresented^26^. Comparison with national data also shows that around 68.5% of mothers and 45.2% of fathers in MoBa aged 25–29 had completed higher education, compared to 47.5% of women and 31.6% of men aged 25–29 in 2005 (the mean year of birth for MoBa children)^27^. Although the figures for Norway also include non-parents, they indicate that parents with more educational qualifications are overrepresented in MoBa. From the whole MoBa cohort, this analysis was restricted to 40,879 mother-father-child trios (Table 1) for whom at least one parental questionnaire had been completed and where genetic information for all three members of the trio was available (numbers excluded for each reason are shown in Supplementary Fig. 1). Comparison based on complete-case data showed that compared to those excluded, mothers and fathers in the analytic sample had, on average, slightly more years of schooling and scored slightly lower for depressive/anxiety traits (all *p* < 0.001; more details are provided in Supplementary Notes). Mothers in the analytic sample scored slightly lower for ADHD, and children in the analytic sample had slightly lower traits of depression, anxiety, and ADHD (all *p* < 0.001). Within the analytic sample, missing values in phenotype data were imputed using multiple imputation. Reflecting longitudinal attrition by the time the children were aged 8 years^28^, there were few missing values for parental characteristics reported during pregnancy (2% or less for mothers’ and fathers’ educational attainment, age, and smoking status) but higher for children’s outcomes measured at age 8 (approximately 54% for depressive, anxiety and ADHD traits; Supplementary Table 2 shows the proportion of values imputed for each variable). The education PGI was a strong instrument even in within-family models. The first-stage F-statistic, which indicates the strength of association between an instrumental variable and an exposure, was 591.6 for mothers and 600.9 for fathers (a first-stage F-statistic greater than ten is generally considered a strong instrument). Meanwhile, the conditional R^2^ showed that the index explained around 1.4% of the variation in years of schooling for both parents.

**Table 1:** Descriptive characteristics of analytic sample (*N* = 40,879)^a^

	Mean	SD	Min	Max
Father’s years of education	14.8	2.6	7.0	21.0
Mother’s years of education	15.4	2.3	8.2^b^	21.0
Mother’s age at child’s birth (years)	30.2	4.4	16.0	46.0
Father’s age (years)	32.6	5.1	18.0	60.0
Mother’s depressive/anxiety traits^c^	1.2	1.9	0.0	15.0
Father’s depressive/anxiety traits^d^	1.1	2.1	0.0	24.0
Mother’s ADHD traits^e^	6.7	3.5	0.0	24.0
Father’s ADHD traits^e^	8.2	3.1	0.0	24.0
Child depressive traits age 8^f^	1.9	2.5	0.0	25.0
Child anxiety traits age 8^g^	1.0	1.2	0.0	10.0
Child ADHD traits (total) age 8^h^	8.6	7.2	0.0	54.0
Child ADHD traits (inattention) age 8^i^	5.0	4.1	0.0	27.0
Child ADHD traits (hyperactivity) age 8^i^	3.6	3.9	0.0	27.0
	Category			%
Child’s sex	Male			51.0
	Female			49.0
Mother’s parity at child’s birth	0			46.8
	1			35.7
	2			14.0
	3			2.7
	4+			0.7
Mother’s marital status at child’s birth	Married/ registered partner			97.4
	Single			2.6
Mother’s smoking status during pregnancy	Never			51.0
	Stopped before week 17			42.0
	Currently			7.0
Father’s smoking status during pregnancy	Never			50.5
	Stopped before week 17			20.8
	Currently			28.6

Descriptive characteristics of the sample used in all analyses. Missing phenotypic data was imputed using multiple imputation by chained equations. Descriptive statistics for the unimputed data are shown in Supplementary Table 1.

^a^The reasons for exclusions and numbers in each case are shown in Supplementary Fig. 1.

^b^This is a non-integer because the values are based on imputed data.

^c^Hopkins Symptoms Checklist based on 5 items, possible range: 0–15.

^d^Hopkins Symptoms Checklist based on 8 items, possible range: 0–24.

^e^Adult ADHD self-report scale, possible range: 0–24.

^f^Short Mood and Feelings Questionnaire, possible range: 0–26.

^g^Screen for Child Anxiety Related Disorders (SCARED), possible range: 0–10.

^h^Parent/Teacher Rating Scale for Disruptive Behaviour Disorders (RS-DBD), possible range: 0–54.

^i^Parent/Teacher Rating Scale for Disruptive Behaviour Disorders (RS-DBD), possible range: 0–27.

### Parental education and childhood traits of depression

**Fig. 2:**
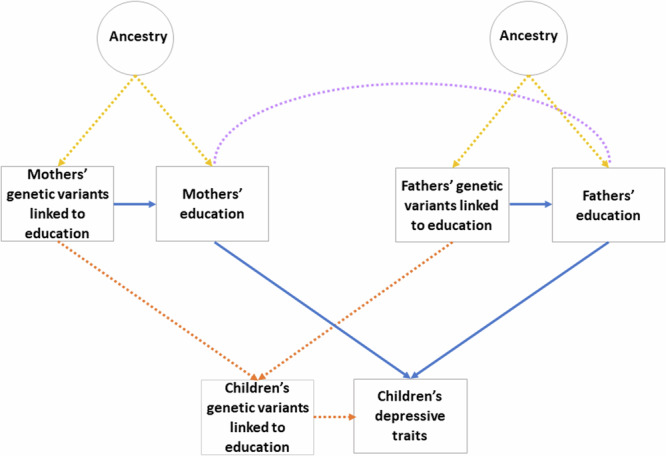
Mendelian randomization applied to an intergenerational relationship: separating co-parental effects. Blue lines: genetic nurture. Orange lines: direct genetic transmission. Lilac lines: assortative mating. Yellow lines: confounding by environmental factors or ancestry. Dotted lines: pathways accounted for by use of instrumental variables or adjustment. To separate maternal from paternal effects, genotype data on both parents as well as children is required. Ancestry can also confound associations of genetic variants with outcomes, so is typically adjusted for via principal components. Adapted from Morris et al.^58^.

**Fig. 3:**
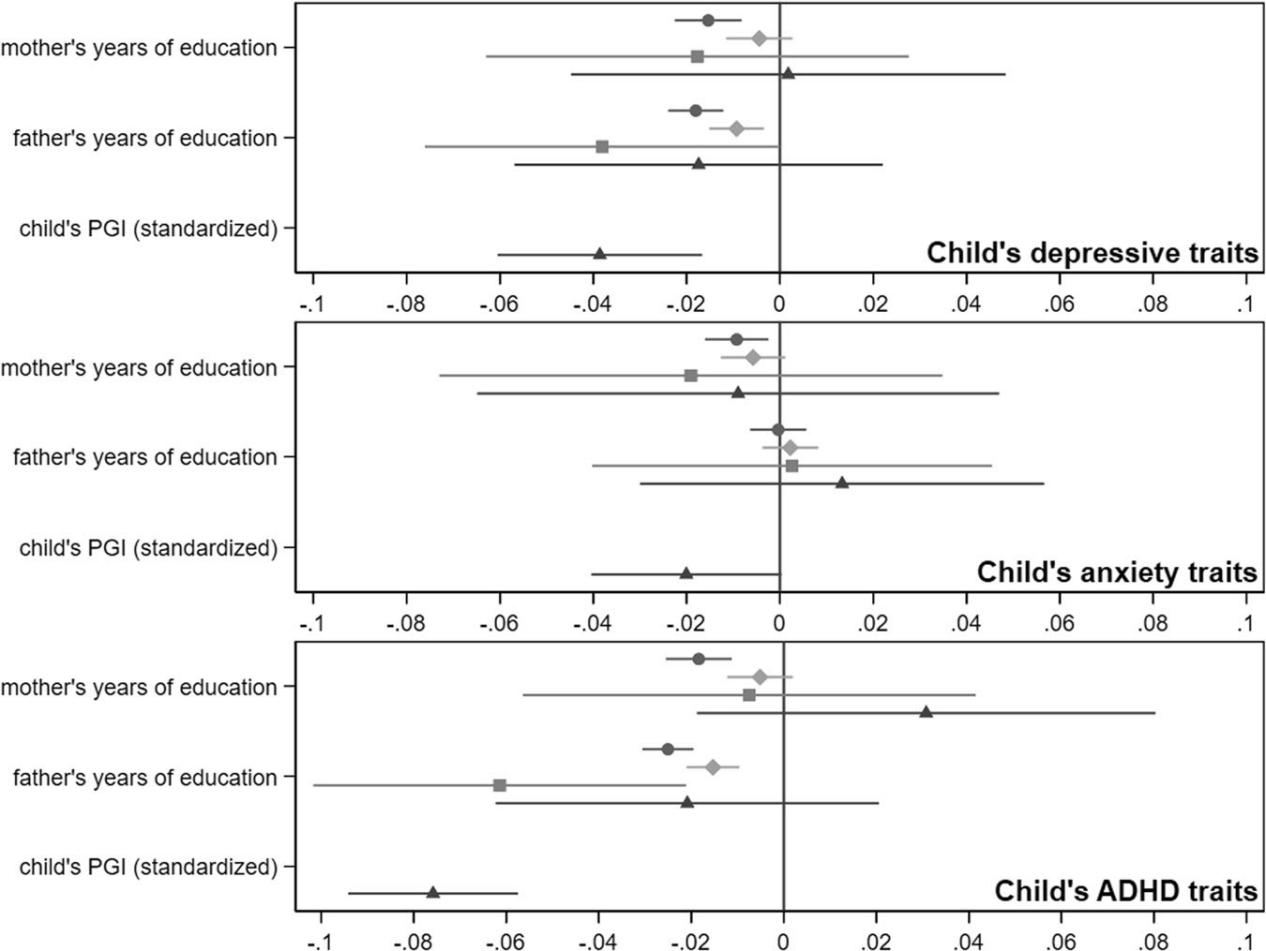
Associations between mother’s and father’s years of education and children’s traits of depression, anxiety, and ADHD, *N* = 40,879. Circles: non-genetic multivariable regression adjusting for the child’s sex, year of birth, and genotyping covariates. Diamonds: non-genetic multivariable regression adjusting for the child’s sex and year of birth, mother’s and father’s traits of depression and ADHD, mother’s and father’s smoking status, maternal parity at the child’s birth, and genotyping covariates. Squares: Mendelian randomization model adjusting for the child’s sex and year of birth, the other parent’s education PGI, and genotyping covariates. Triangles: within-family Mendelian randomization, adjusting for the child’s own education PGI, sex and year of birth, the other parent’s education PGI, and genotyping covariates. All outcomes are standardized; mother’s and father’s education are in years.

Results from main models are shown in Fig. 2 with coefficients, CIs and p values shown in Supplementary Table 3. In non-genetic models adjusting only for the child’s sex, year of birth, and genotyping covariates, there were small but significant associations between both parents’ years of education and children’s depressive traits, which decreased by 0.02 S.D. (CI = 0.01, 0.02, *p* < 0.001) per year of education for both parents. Adjusting for likely confounders, an extra year of paternal education was associated with a 0.01 (CI = 0.02, 0.00, *p* = 0.002) decrease in children’s depressive traits, but the maternal effect was consistent with the null (beta = 0.00, CI = −0.01, 0.00, *p* = 0.22). Estimates from Mendelian randomization which did not control for child genotype were similar, but less precise (maternal: −0.02, CI = −0.06,0.03, *p* = 0.44, paternal: −0.04, −0.08,0.00, *p* = 0.05). In Mendelian randomization models which also controlled for the child’s genotype, there was little evidence that either parent’s education affected their children’s outcomes, although the confidence intervals were wide (maternal: 0.00, CI = −0.04, 0.05, *p* = 0.94, paternal: −0.02, CI = −0.06, 0.02, *p* = 0.39). However, the child’s own PGI for educational attainment negatively associated with their depressive traits. The effect was small: a one-S.D. change in the PGI corresponded to a decrease of 0.04 S.D. (CI = 0.02, −0.02, *p* = 0.001) in depressive traits, or around 0.1 point on a 26-point scale (Fig. 3).

### Parental education and childhood traits of anxiety

In non-genetic models adjusting only for the child’s sex, year of birth, and genotyping covariates, an extra year of maternal education corresponded to a 0.01 S.D. (CI = 0.00,0.02, *p* = 0.008) decrease in children’s anxiety traits. This attenuated with adjustment for likely confounders (to 0.01 S.D., CI = 0.00,0.01, *p* = 0.10), while fathers’ education was not associated with children’s anxiety traits in any model. The Mendelian randomization estimates were imprecise, but provided little evidence of an effect of either parent’s education. There was some evidence that the child’s own PGI for educational attainment affected anxiety traits, but the effect was small: per S.D. change in the PGI, anxiety traits were 0.02 S.D. lower (CI = −0.00, 0.04, *p* = 0.05), or 0.02 points on a 10-point scale.

### Parental education and childhood traits of ADHD

In non-genetic models adjusting only for the child’s sex, year of birth, and genotyping covariates, both mothers’ and fathers’ education was negatively associated with children’s ADHD traits: children’s ADHD traits were 0.02 S.D. (CI = 0.01,0.03, *p* < 0.001) lower per year of maternal education, and 0.03 S.D. (CI = 0.02,0.03, *p* < 0.001) lower per year of paternal education. Adjustment for confounders reduced these differences (e.g., for maternal education, beta = −0.01 S.D. (−0.01,0.00,*p* = 0.15). In Mendelian randomization models which did not control for children’s genotype, the estimated effect of paternal education was larger than in non-genetic models (−0.06, CI: −0.10, −0.02, *p* = 0.003), but this attenuated with adjustment for the child’s PGI (−0.02, CI: −0.06, 0.02). There was little evidence from either genetic model that mothers’ education was associated with the child’s ADHD traits. However, the child’s own PGI for educational attainment was negatively associated. A one-S.D. increase in the PGI corresponded to ADHD traits 0.08 S.D. lower (CI = 0.06, 0.09, *p* < 0.001), a difference of 0.6 points on a 54-point scale. Results for ADHD subscales were consistent with results for the full scale (Supplementary Table 3).

### Sensitivity analyses

Sex-specific models were broadly consistent with the main results, although less precise (females: Supplementary Fig. 2 and Supplementary Table 4, males: Supplementary Fig. 3 and Supplementary Table 5). Results from models using square root-transformed version of the outcomes (Supplementary Fig. 4, Supplementary Table 6) were consistent with main results. Results based on unimputed data in the much smaller complete-case sample for each outcome were less precise (Supplementary Fig. 5, Supplementary Table 7). The child’s own PGI for educational attainment was clearly associated with their ADHD traits (−0.06, CI: −0.10, −0.03, *p* = 0.001) with similar associations for the subscales, but not with their anxiety or depressive traits. Unexpectedly, in Mendelian randomization models adjusting for the child’s genotype, mother’s educational attainment was positively related to the child’s depressive (0.16, CI: 0.02, 0.30, *p* = 0.03) and ADHD-inattention traits (0.21, CI: 0.06, 0.35, *p* = 0.006).

### Two-sample summary data Mendelian randomization

Results from two-sample MR are presented in Supplementary Tables S8 and S9. Using all SNPs included in the PGI (Supplementary Table 8), inverse variance weighted (IVW) regression implied that more years of maternal education were related to fewer depressive traits (−0.04, CI: −0.08, −0.01) and ADHD-hyperactivity traits (−0.04, CI: −0.08, 0.00), but more ADHD-inattention traits (0.06, CI: 0.02, 0.10) in children. IVW models implied that more years of paternal education was related to fewer depressive traits (−0.06, CI: −0.10, −0.03) and ADHD traits (−0.04, CI: −0.07, 0.00) in children, with similar associations for the inattention and hyperactivity subscales. Estimates from MR-Egger, MR-Median, and MR-Modal were consistent with the null. There was little evidence of pleiotropy from the MR-Egger intercept, which crossed the null for all outcomes. Using a subset of 510 SNPs clumped to a more stringent independence threshold, estimates were less precise but consistent with estimates based on all SNPs in the PGI (Supplementary Table 9). The only estimate distinguishable from the null was from IVW, suggesting a positive effect of maternal education on ADHD-inattention traits (0.07, CI: 0.02, 0.13).

## Discussion

In a large sample of Norwegian 8-year-olds and their parents, children born to parents with more years of education tended to score slightly lower for depressive and ADHD traits. Children born to mothers with more years of education also tended to score slightly lower for anxiety traits. However, these differences reduced when confounders were accounted for, and estimates from within-family MR models, which are less affected by confounding by environmental factors, found little evidence that parents’ educational attainment causally impacted their children’s depressive, anxiety, or ADHD traits. Although genetic estimates were imprecise, we were able to show that effects larger than 0.08 S.D. per year of parental education on these outcomes are unlikely. In additional analyses, a positive association from inverse-variance weighted regression of mothers’ education with children’s ADHD-inattention traits only may reflect a type 1 error and should be interpreted in the context of overall findings. Positive associations between mother’s educational attainment and children’s ADHD-inattention and depressive traits were also seen in within-family MR models based on complete-case data. Again, these should be interpreted in the context of overall findings. Since these were restricted to around 15% of the analytic sample with no missing data, complete-case estimates are likely to be affected by additional biases related to selection^28,29^, as well as being imprecise. Interestingly, children’s own PGI for educational attainment were associated with fewer traits of depression and ADHD, independent of their parents’ PGIs, although effect sizes were small. This suggests that the correlation between parents’ educational attainment and children’s depressive and ADHD traits may be driven more by direct genetic effects than by genetic nurture effects. Together, these results suggest that children’s traits of depression, anxiety, inattention or hyperactivity are unlikely to be causally linked to variation in parental education - at least in societies where most younger adults already participate in tertiary education^30^. Consequently, other policies will be needed to target children’s emotional and behavioural difficulties despite increasing levels of educational attainment in many countries. This contrasts with non-genetic studies from Spain^6^, Germany^7^ Finland^8^ and Norway^9^ which report robust associations of parental education and children’s mental health, suggesting that increases to adult education may benefit children as well as their parents. The discrepancy may reflect heterogeneity in relationships by country context, differences in the methods used, or use in previous studies of more nationally representative study populations including more parents with fewer qualifications.

In line with the current study, a Dutch study found that children’s ADHD was predicted by their own polygenic indices for educational attainment but found little evidence for an influence of parents via genetic nurture effects^20^. Two studies using a subset of the current cohort used a genetic family design to estimate overall genetic nurture effects (i.e., the total influence of parents’ genotypes mediated by any parental phenotype, as opposed to the influence mediated by specific parental traits of interest) on children’s depressive, anxiety^17^ and ADHD traits^18^. This found clearer evidence of genetic nurture for depression and ADHD than for anxiety. However, maternal and paternal effects could not be separated, and the role of specific parental traits (for example, parental educational attainment) was not explored. Our results suggest parental educational attainment is unlikely to substantially account for those effects, and that other parental characteristics may be more important for these outcomes. Our results accord with a similar study using 19,506 trios from an earlier, smaller release of the MoBa genetic data^19^. That study found clear evidence that children’s own education PGIs influence their ADHD traits, but little evidence for genetic nurture effects, and we find similar relationships in a much larger group of genotyped trios. Our findings partly accord with a study which applied an extended children-of-twins design in MoBa to examine effects of parental education on children’s depressive and ADHD traits^16^. As in our study, associations with depressive traits were fully explained by shared familial risk factors, but associations with ADHD traits were not fully explained by these factors, suggesting genetic nurture effects of parents’ education on children’s ADHD traits. However, these effects were small, and were consistent with our within-family MR results, falling within our estimates’ confidence limits. The greater precision of estimates from the earlier study reflects the fact that approaches such as children-of-twins studies, which are based on variance decomposition, consider all additive genetic liability in a trait. In contrast, approaches using PGIs can only consider genetic liability associated with specific SNPs^31^. Confidence intervals of our within-family MR estimates for ADHD are therefore consistent with small causal effects.

Our results for parental education contrast with several studies which find evidence that children’s emotional and behavioural difficulties are influenced by parental income^9,32,33^. It may indeed be that parental income has greater effects on children’s emotional and behavioural difficulties than parental education. It may also be that the mental health consequences of social disadvantage are more visible in contexts with larger socioeconomic disparities than in comparatively equal societies, including present-day Norway. In contrast, other work using this cohort suggests that relatives’ education has substantial effects on children’s educational outcomes, including results in national tests^34–36^. This indicates that aspects of the family environment linked to parental education may influence children’s educational outcomes more than their mental health or behavioural outcomes. Additionally, outcomes in the current study were based on mothers’ assessments of children’s traits, which could have biased estimates. Follow-up analyses could examine effects of parental education on children’s diagnoses based on medical records. However, diagnostic biases may also distort inequalities in these outcomes when assessed via diagnoses^37^. Associations may depend on the age group: other evidence from Norway points to a possible impact of parental education on emotional and behavioural difficulties of adolescents^38^.

Although effect sizes were small, our results suggest that a child’s own genetic liability for educational attainment may have a protective effect on both depression and ADHD traits at age 8. Findings accord with previous work suggesting an influence of genetic liability for educational attainment on ADHD traits^20,39–41^. Caution is warranted when interpreting such results, since in this age group, differences in educational attainment itself are yet to emerge. This suggests the effect of genetic liability for educational attainment on children’s depression and ADHD traits at this age may be driven by other phenotypes related to educational attainment. Related phenotypes could include other aspects of mental and somatic health, cognitive traits, or non-cognitive traits such as personality or behaviour^42^. Alternatively, this may reflect overlapping genetic architecture of educational attainment with that of depressive and ADHD traits^43^: to the extent that these traits influence educational attainment in adulthood, our educational attainment PGI may also capture genetic variation linked to these traits. Follow-up work using multivariable MR to separate the influence of closely related traits could be applied to disentangle these processes. Importantly, the effect of genetic variants linked to educational attainment on a child’s depressive or ADHD traits is likely to depend on the child’s home and school environment. Other research using MoBa has shown that the consequences of genetic predispositions vary between environments: children with similarly low values of a polygenic index for educational attainment do better in national tests in some schools than others, even after accounting for family characteristics^44^. Such findings make clear that a child’s polygenic index for educational attainment may influence their depressive or ADHD traits more in some circumstances than others, and further research is needed to identify how students of all abilities can be best supported. For example, evidence suggests that a greater sense of connectedness to school and enjoyment of school may be beneficial for children’s depressive and externalizing difficulties^45^. These are likely to be modifiable, given that students’ sense of belonging at school differs substantially across countries and, within countries including Norway, according to how cooperative students perceive their school to be^46^.

A key strength of this study is that we used one of the largest existing samples of genotyped mother-father-child trios to explore the effects of maternal and paternal educational on children’s emotional and behavioural traits. We applied a genetic approach which is robust to environmental confounding, whilst controlling for effects due to direct inheritance. Nevertheless, genetic results were imprecise, and larger samples of genotyped trios will be needed to fully delineate effects. Selection both into the MoBa sample (whose participation rate was 41%), and into the subsample of genotyped trios (around 36% of the full MoBa cohort) meant that parents included in analyses were, on average, more highly educated and in better health than the Norwegian population as whole. This may have influenced findings and the generalizability of conclusions to the Norwegian population. At the same time, education-related differences in mental health are likely contingent on a country’s education system, health system, and overall levels of social inequality. It is therefore possible that different associations with parental educational attainment would be observed in other countries. Within the analytic sample, there was a considerable proportion of missing values in outcomes, and imputation may not have fully recovered the distribution of missing values. However, estimates from complete-case analysis of longitudinal data can be substantially biased due to differential attrition, which has been shown to occur in this cohort^28^. Use of parent-reported outcomes, which are subject to reporting biases, may also have influenced results. Further work is needed in larger and more representative studies with family-based genetic data, among children in different age groups, and in countries with different school systems and levels of educational and economic inequality.

In a large Norwegian general population survey, any causal effects of maternal and paternal educational attainment on children’s emotional and behavioural traits are likely to be modest compared to other aspects of the child’s environment. In contrast, children’s traits or behaviours related to later educational attainment may positively influence their emotional and behavioural outcomes. Further work is needed in larger and more representative family-based studies and in countries with different education systems and levels of social inequality.

## Methods

### Study population

The Norwegian Mother, Father and Child Cohort Study (MoBa) is a population-based pregnancy cohort study conducted by the Norwegian Institute of Public Health^25^. Participants were recruited from all over Norway from 1999 to 2008. The women consented to participation in 41% of the pregnancies. The cohort includes approximately 114,500 children, 95,200 mothers and 75,200 fathers. All the mothers and fathers provided written informed consent at recruitment during the pregnancy. Blood samples were obtained from both parents during pregnancy and from mothers and children (umbilical cord) at birth^47^. The first child was born in October 1999 and the last in July 2009. The current study is based on version 12 of the quality-assured data files released for research in January 2019. The establishment of MoBa and initial data collection was based on a license from the Norwegian Data Protection Agency and approval from The Regional Committees for Medical and Health Research Ethics. The MoBa cohort is currently regulated by the Norwegian Health Registry Act. The current study was approved by The Regional Committees for Medical and Health Research Ethics (2016/1702). The Medical Birth Registry (MBRN) is a national health registry containing information about all births in Norway. This analysis was restricted to 40,879 mother-father-child trios for whom genetic data were available for all three individuals, and where the mother had completed at least one questionnaire between pregnancy and when the child was aged 8 which could be used in imputation.

The numbers of participants excluded are shown in a STROBE flow chart in Supplementary Fig. 1. From all records in MoBa (*N* = 113,603, after removing consent withdrawals), participants were excluded if the parents had not completed any of the MoBa questionnaires used in imputation models. Of 104,723 records remaining, there were 40,879 births for which genetic data were available and had passed QC filters for mother, father, and child. Full details of the genotyping and genetic quality control are provided elsewhere^48^. Related participants were retained, but all models were clustered by genetic family ID derived using KING software^49^. This genetic family ID groups first, second, and third-degree relatives (i.e., siblings in the parental generation and their children as well as nuclear families), in this way accounting for non-independence of observations.

### Measures

Information on parents’ educational attainment came from linked administrative data from Statistics Norway, available for 99% of parents. Mothers’ and fathers’ highest educational qualification as reported in questionnaires was used to impute the small number of missing values. We converted parents’ responses to years of education according to the International Standard Classification of Education (ISCED) categories for education, as has been used for previous studies. Details of this mapping is described elsewhere^36^.

The child’s depressive, anxiety, and ADHD traits were reported by the mother when the child was 8 years old using validated measures. For depressive traits, the 13-item Short Mood and Feelings Questionnaire (SMFQ) was used, for anxiety traits the 5-item Short Screen for Child Anxiety Related Disorders (SCARED)^50^ and for ADHD traits the Parent/Teacher Rating Scale for Disruptive Behaviour Disorders (RS-DBD) (total score and subdomain scores for inattention and hyperactivity)^51^. Prorated summary scores were calculated for individuals with at least 80% of item-level information. Mothers’ and fathers’ depressive/anxiety traits were self-reported using selected items from the 25-item Hopkins Checklist^52^ and mother’s and father’s ADHD traits (from the 6-item adult ADHD self-report scale^53^. Full details of all questions asked in MoBa are available at https://www.fhi.no/en/ch/studies/moba/for-forskere-artikler/questionnaires-from-moba/.

Polygenic indexes (PGI) for educational attainment were calculated using single-nucleotide polymorphisms (SNPs) associated at genome-wide significance (*p* < 5.0 × 10^−8^) with years of schooling in the largest and most recent GWAS of educational attainment^13^ and weighted using the individual SNP-coefficients from the GWAS. We used GWAS results based on all study samples including 23andme, available from the Social Science Genomics Consortium https://thessgac.com/papers/14. We excluded SNPs not available in MoBa, and then used the TwoSampleMR package^54^ to identify 1729 SNPs independently associated with educational attainment at *p* < 5.0 × 10^−8^ (with a clumping threshold of r^2^ = 0.01,  window = 10,000 kb).

### Multiple imputation of phenotype data

Multiple imputation by chained equations was performed in STATAv16 to estimate missing phenotypic information for the 40,879 trios with complete genetic data. 50 imputed datasets were produced and analysis across these datasets conducted with STATA’s mi estimate commands. The imputation model included mother’s and father’s years of education, all outcome variables used in the main analyses, the child’s sex and year of birth, and other phenotypic covariates used in non-genetic models, including mother’s and father’s smoking status reported at approximately 17 weeks gestation, mother’s and father’s depressive/anxiety traits (using selected items from the 25-item Hopkins Checklist^52^), and ADHD traits (from the 6-item adult ADHD self-report scale^53^), maternal parity at the child’s birth. Variables from the birth registry file were also included as auxiliary variables: the mother’s marital status, number of previous pregnancies, and the age of the mother and father. Ordered categorical variables were imputed with ordered logistic regression. There was no missingness in genetic information within the analytic sample. Polygenic scores were included on the right-hand side of the imputation equations, along with indicators for genotyping centre and chip and the first 20 principal components of ancestry for all individuals. Continuous variables were imputed with predictive mean matching, specifying knn(10). This included child’s depressive and anxiety traits at age 8 (SMFQ and SCARED summary scores), mother’s and father’s depressive/anxiety traits at 17 weeks gestation (summary scores based on items from the 25-item Hopkins Checklist^52^), and mother’s and father’s ADHD traits from the 6-item adult ADHD self-report scale^53^. To facilitate analysis of ADHD inattention and hyperactivity subscales, the two subscales were imputed, again with predictive mean matching, and the full scale calculated post-imputation with mi passive. An earlier measure of the child’s ADHD traits at 5 years, based on questions from the Short-Form Conners Parent Rating Scale^55^, was included as an auxiliary variable. The % of imputed data in the analytic sample was lowest for maternal characteristics like marital status and parity at the child’s birth (0.0%) and highest for father’s ADHD traits (60.0%); more information is given in Supplementary Table 2.

### Statistical analysis

First, we regressed depressive, anxiety, and ADHD traits, and subdimensions of ADHD (inattention and hyperactivity) on maternal and paternal education, adjusting for the child’s sex and birth year. For comparability, these models also included all covariates included in genetic models: genotyping centre, genotyping chip, and 20 principal components of ancestry for the child, mother, and father. Second, we regressed the same outcomes on maternal and paternal education, with further adjustment for likely confounders of observational associations: mothers’ and fathers’ depressive/anxiety traits and ADHD traits, mothers’ and fathers’ smoking status during pregnancy, and maternal parity at the child’s birth. Third, we ran Mendelian randomization models for each outcome in which the mothers’ and fathers’ PGIs were used to instrument the mothers’ and fathers’ educational attainment. These were adjusted for the child’s sex and birth year, the other parent’s PGI, and genotyping covariates. Fourthly, we conducted within-family MR models. These were multivariable MR models, in which the mothers’ and fathers’ PGIs were used to instrument the mothers’ and fathers’ educational attainment, and the child’s PGI included as a covariate to account for direct genetic effects. These models also adjusted for the child’s sex and year of birth, and genotyping covariates. Within-family MR models were conducted with two-stage least squares instrumental-variable regression using Stata’s ivregress, with F-statistics and R^2^ values estimating the instrument’s strength obtained using ivreg2. All models used robust standard errors (Stata’s vce option).

### Sensitivity analyses

To investigate for sex-specific maternal and paternal effects, we ran imputation and analysis models stratified by the sex of the child. To check the sensitivity of results to distributional assumptions, analyses were repeated using square root-transformed versions of outcome measures, which may be preferable to log-transformation when distributions include zero values^56^. For completeness, we repeated analysis using complete-case (unimputed) data on trios with full genetic, exposure, outcome, and covariate data (complete-case *N* = 6295 for depressive traits, *N* = 6311 for anxiety traits, and *N* = 6308 for ADHD traits).

### Two-sample summary data Mendelian randomization

Genetic studies designed to assess causation can be biased by horizontal pleiotropy^21^. This is when genetic variants in a PGI influence the outcome via pathways which do not involve the exposure. In intergenerational MR studies, two types of horizontal pleiotropy can occur. First, SNPs used to instrument parental exposures can have independent effects when transmitted to children. Conditioning on the child’s genotype for the same SNPs blocks this path and removes this source of bias. However, estimates of the effects of specific parental exposures can still be affected by pleiotropy if SNPs used to instrument a parental exposure influence other relevant features of the parent. For instance, SNPs linked to parental education may also affect parental depression, plausibly affecting children’s emotional and behavioural difficulties independently of their parents’ education. Methods have been developed to test for the presence of horizontal pleiotropy by comparing SNP-specific associations of exposures and outcomes, which themselves rest on assumptions^24^. We therefore carried out additional analyses using SNP-level associations for the SNPs included in the PGI. With these methods, non-independence of SNPs can lead to overestimation of the precision of estimates. We therefore ran these analyses twice: using all 1729 SNPs in the educational attainment PGI and using a subset of 510 SNPs which were independent at a more stringent clumping threshold of r^2^ = 0.001. Associations with parental educational attainment came from the GWAS, and we estimated SNP-level associations with children’s outcomes in MoBa using linear models which mutually adjusted for mother, father and children’s genotypes and covariates used in within-family MR models. It was not computationally feasible to include individual SNPs in the imputation models, so SNP-outcome associations in MoBa were calculated using unimputed SNP data with imputed outcome data. We conducted inverse-variance weighted, MR-Median, MR-Mode, and MR-Egger regression in STATAv16 with the MRRobust package^57^. An MR-Egger intercept distinguishable from the null indicates presence of heterogeneity across the variants, which is consistent with horizontal pleiotropy.

### Reporting summary

Further information on research design is available in the Nature Research Reporting Summary linked to this article.

### Supplementary information

Supplementary material

Reporting Summary

## Data Availability

MoBa data is not publicly available because the consent given by the participants does not allow for data storage on an individual level in repositories or journals. Researchers who want access to data sets for replication should apply to datatilgang@fhi.no. Access to data sets requires approval from The Regional Committee for Medical and Health Research Ethics in Norway and an agreement with MoBa.

## Code availability

Code used to generate these results is available at https://github.com/ammh/moba_parent_education_dep_anx_adhd.
