# Supplementary material for: Parental education and children’s depression, anxiety, and ADHD traits, a within-family study in MoBa
Source: NPJ Sci Learn. 2024 Jul 18;9:46. doi: 10.1038/s41539-024-00260-8 (PMC11258307; doi:10.1038/s41539-024-00260-8)
Supplement: Supplementary file 1 — Supplementary material [file 41539_2024_260_MOESM1_ESM.pdf]

Supplementary material: Intergenerational associations between education and traits of depression, anxiety and ADHD: a within-family Mendelian randomization in MoBa

Supplementary figures:

Supplementary Figure 1: Flow chart of inclusion and exclusion of MoBa participants into the study sample

Supplementary Figure 2: Associations between mother's and father's years of education and children's traits of depression, anxiety, and ADHD, female children only (N=20,013)

Supplementary Figure 3: Associations between mother's and father's years of education and children's traits of depression, anxiety, and ADHD, male children only (N=20,866)

Supplementary Figure 4: Associations between mother's and father's years of education and children's square root-transformed traits of depression, anxiety, and ADHD (N=40,879)

Supplementary Figure 5: Associations between mother's and father's years of education and children's traits of depression anxiety and ADHD, complete-case data

Supplementary tables:

Supplementary Table 1: Descriptive characteristics of analytic sample, unimputed data

Supplementary Table 2: Proportion of imputed data for all variables in analytic dataset

Supplementary Table 3: Parental education and children's traits of depression anxiety and ADHD

Supplementary Table 4: Parental education and children's traits of depression anxiety and ADHD, females only

Supplementary Table 5: Parental education and children's traits of depression anxiety and ADHD, males only

Supplementary Table 6: Parental education and children's square root-transformed traits of depression anxiety and ADHD

Supplementary Table 7: Parental education and children's traits of depression anxiety and ADHD, complete-case analysis

Supplementary Table 8: Results of two-sample summary data Mendelian randomization, 1729 SNPs in educational attainment polygenic index

Supplementary Table 9: Results of two-sample summary data Mendelian randomization, 510 SNPs in educational attainment polygenic index

Supplementary notes:

Supplementary Note 1: Comparison of included and excluded participants

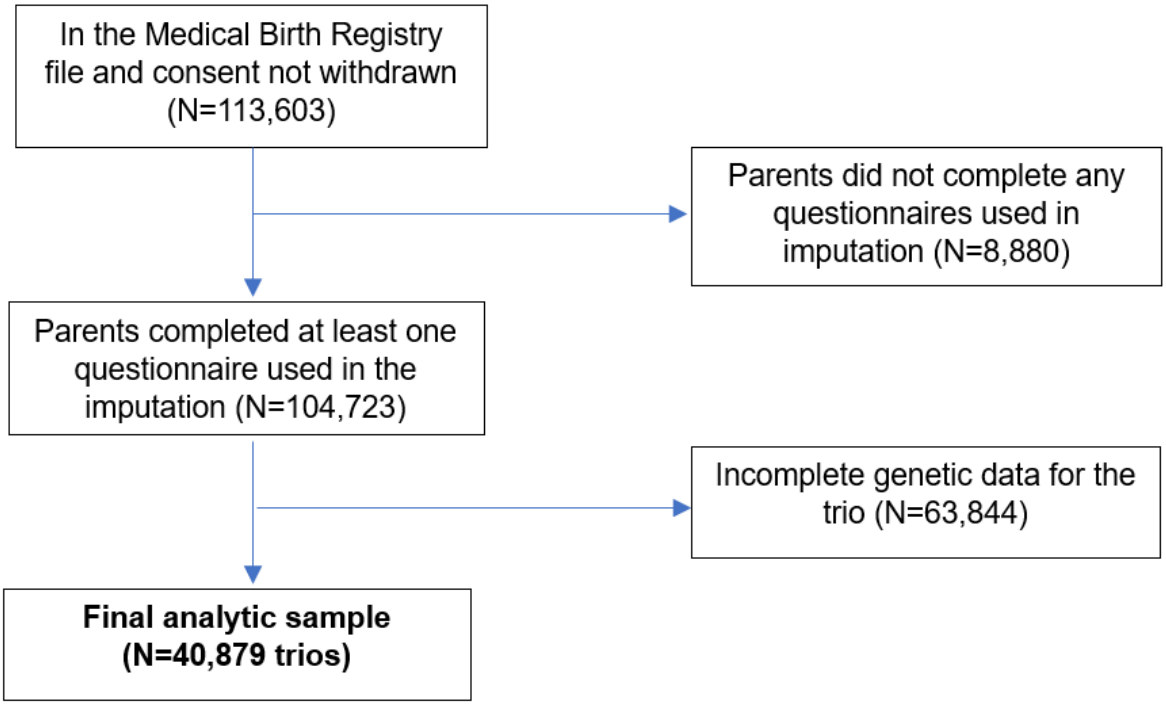

Supplementary Figure 1: Flow chart of inclusion and exclusion of MoBa participants into the study sample. We began with all MoBa pregnancies with a record from the Medical Birth Registry of Norway (excluding consent withdrawals). From these, we excluded trios where the parents had not completed any questionnaires used in imputation of phenotype data, and where genetic data was not available for all members of the trio. This left a final analytic sample of 40,879 trios.

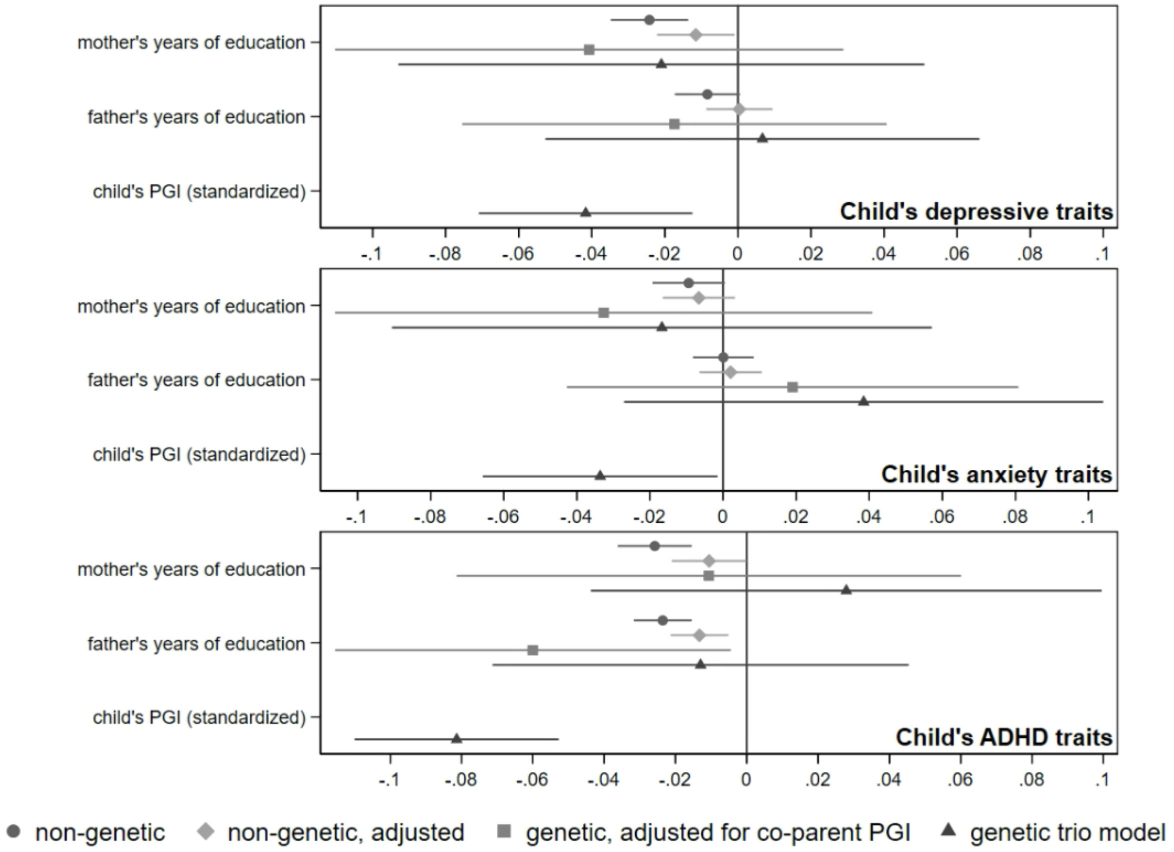

Supplementary Figure 2: Associations between mother's and father's years of education and children's traits of depression, anxiety, and ADHD, female children only (N=20,013). Non-genetic model: multivariable regression adjusting for the child's year of birth, and genotyping covariates. Non-genetic, adjusted model: multivariable regression adjusting for the child's year of birth, mother's and father's traits of depression and ADHD, mother's and father's smoking status, maternal parity at the child's birth, and genotyping covariates. Genetic, adjusted for co-parent's PGI: Mendelian randomization model adjusting for the child's year of birth, the other parent's education PGI, and genotyping covariates. Genetic trio model: within-family Mendelian randomization, adjusting for the child's own education PGI and year of birth, the other parent's education PGI, and genotyping covariates. All outcomes are standardized; mother's and father's education are in years.

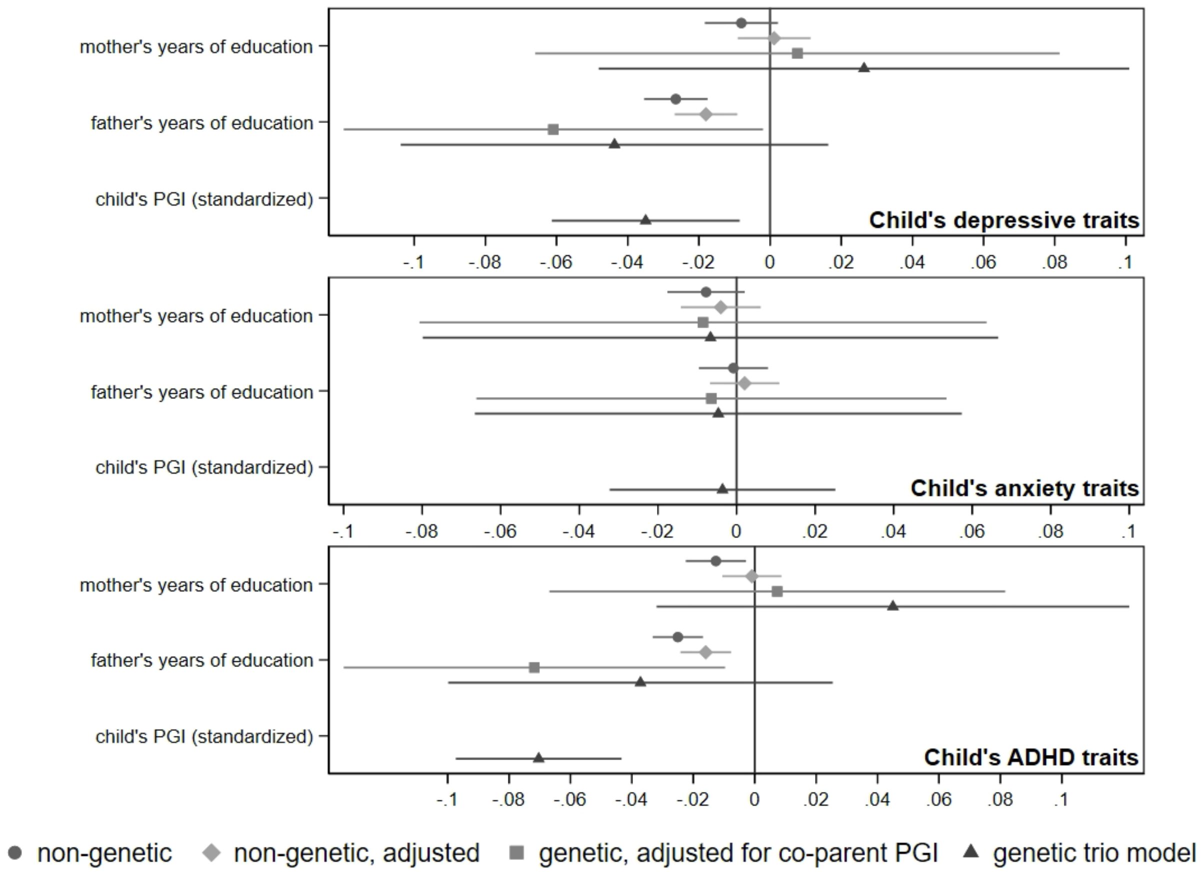

Supplementary Figure 3: Associations between mother's and father's years of education and children's traits of depression, anxiety, and ADHD, male children only (N=20,866). Non-genetic model: multivariable regression adjusting for the child's year of birth, and genotyping covariates. Non-genetic, adjusted model: multivariable regression adjusting for the child's year of birth, mother's and father's traits of depression and ADHD, mother's and father's smoking status, maternal parity at the child's birth, and genotyping covariates. Genetic, adjusted for co-parent's PGI: Mendelian randomization model adjusting for the child's year of birth, the other parent's education PGI, and genotyping covariates. Genetic trio model: within-family Mendelian randomization, adjusting for the child's own education PGI and year of birth, the other parent's education PGI, and genotyping covariates. All outcomes are standardized; mother's and father's education are in years.

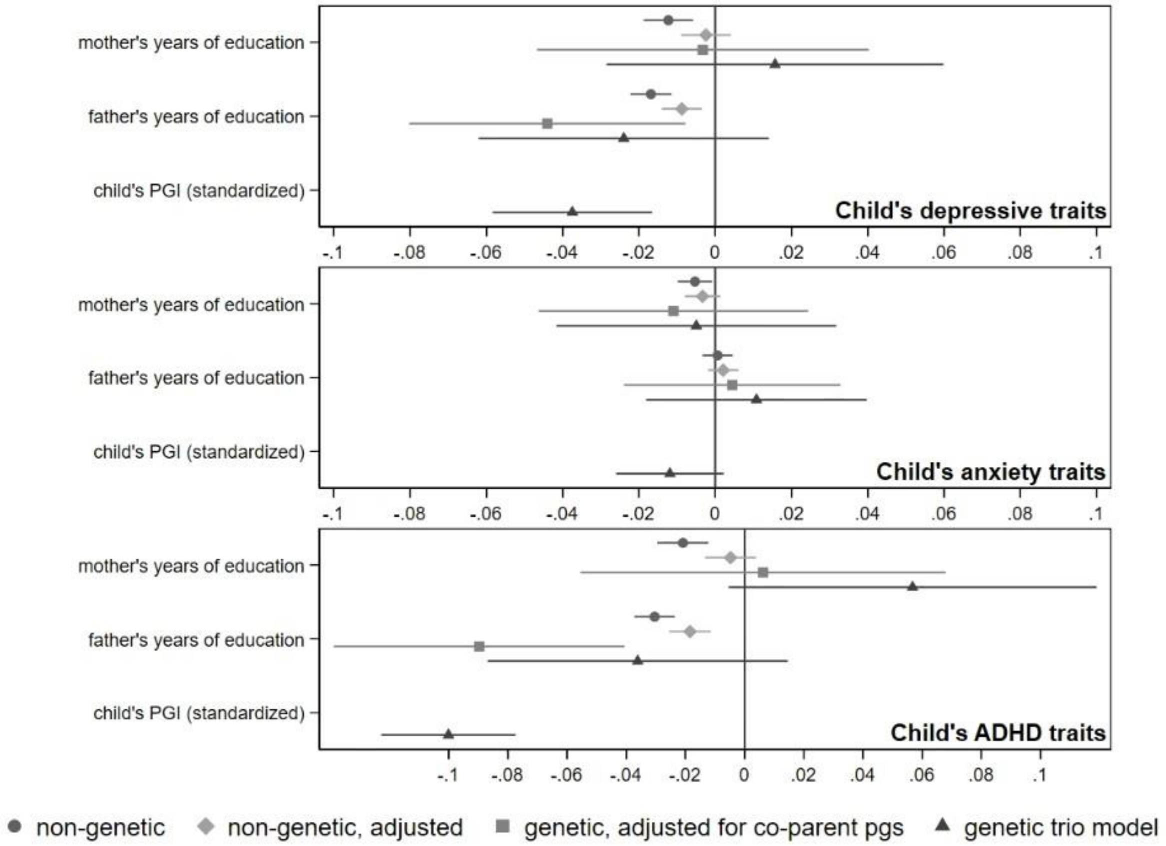

Supplementary Figure 4: Associations between mother's and father's years of education and children's square root-transformed traits of depression, anxiety, and ADHD (N=40,879). Non-genetic model: multivariable regression adjusting for the child's sex and year of birth, and genotyping covariates. Non-genetic, adjusted model: multivariable regression adjusting for the child's sex and year of birth, mother's and father's traits of depression and ADHD, mother's and father's smoking status, maternal parity at the child's birth, and genotyping covariates. Genetic, adjusted for co-parent's PGI: Mendelian randomization model adjusting for the child's sex and year of birth, the other parent's education PGI, and genotyping covariates. Genetic trio model: within-family Mendelian randomization, adjusting for the child's own education PGI, sex and year of birth, the other parent's education PGI, and genotyping covariates. All outcomes are standardized; mother's and father's education are in years.

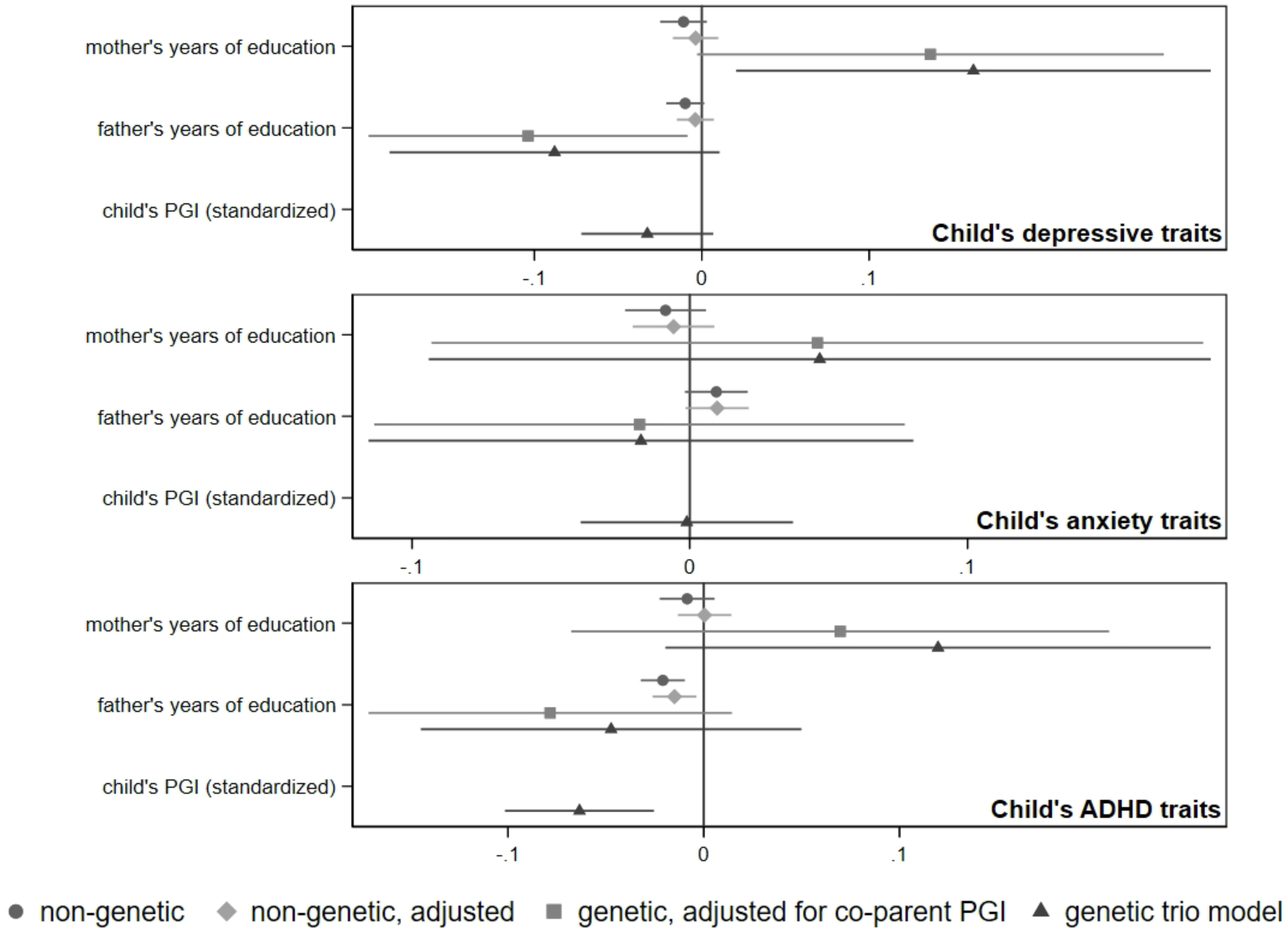

Supplementary Figure 5: Associations between mother's and father's years of education and children's traits of depression anxiety and ADHD, complete-case data. N=6,295 (depressive traits), N=6,311 (anxiety traits), N=6,302 (ADHD traits). Non-genetic model: multivariable regression adjusting for the child's sex and year of birth, and genotyping covariates. Non-genetic, adjusted model: multivariable regression adjusting for the child's sex and year of birth, mother's and father's traits of depression and ADHD, mother's and father's smoking status, maternal parity at the child's birth, and genotyping covariates. Genetic, adjusted for co-parent's PGI: Mendelian randomization model adjusting for the child's sex and year of birth, the other parent's education PGI, and genotyping covariates. Genetic trio model: within-family Mendelian randomization, adjusting for the child's own education PGI, sex and year of birth, the other parent's education PGI, and genotyping covariates. All outcomes are standardized; mother's and father's education are in years.

Supplementary Table 1: Descriptive Characteristics of Analytic Sample, unimputed data[a]			
	mean	SD	N
Father’s years of education	14.8	2.6	40502
Mother’s years of education	15.4	2.3	40570
Mother’s age (years)	30.2	4.4	40879
Father’s age (years)	32.6	5.1	40875
Maternal depressive/anxiety traits, Hopkins Symptoms Checklist-25 (SCL-25)[b]	1.2	1.9	39601
Paternal depressive/anxiety traits, Hopkins Symptoms Checklist-25 (SCL-25)[c]	1.1	2.1	38006
Maternal ADHD traits: adult ADHD self-report scale[d]	6.5	3.4	24166
Paternal ADHD traits: adult ADHD self-report scale[e]	8.2	3.1	16335
Child depressive traits age 8: Short Mood and Feelings Questionnaire (SMFQ)[f]	1.8	2.4	18785
Child anxiety traits age 8: Screen for Child Anxiety Related Disorders (SCARED)[g]	1.0	1.2	18816
Child ADHD traits age 8: Parent/Teacher Rating Scale for Disruptive Behaviour Disorders (RS-DBD)[h]	8.4	7.1	18795
Child ADHD traits (inattention) age 8: Parent/Teacher Rating Scale for Disruptive Behaviour Disorders (RS-DBD)[i]	4.9	4.0	18776
Child ADHD traits (hyperactivity) age 8: Parent/Teacher Rating Scale for Disruptive Behaviour Disorders (RS-DBD)[j]	3.5	3.8	18769
	category	%	
Child’s sex	male	51.0	40879
	female	49.0	
Mother’s parity at child’s birth	0	46.8	40879
	1	35.7	
	2	14.0	
	3	2.7	
	4+	0.7	
Mother’s marital status at child’s birth	married/ registered partner	97.4	40879
	single	2.6	
Mother’s smoking status during pregnancy	never	51.1	40071
	stopped before week 17	42.0	
	currently	6.9	
Father’s smoking status during pregnancy	never	50.4	40598
	stopped before week 17	20.9	
	currently	28.7	

[a]Descriptive statistics for imputed data are shown Table 1. [b]Based on 5 items. Possible range: 0-15. [c]Based on 8 items. Possible range: 0-24. [d]Possible range: 0-24. [e]Possible range: 0-24. [f]Possible range: 0-10. [g]Possible range: 0-54. [h]Possible range: 0-27. [i]Possible range: 0-27.

**Supplementary Table 2: Proportion of imputed data for key variables in analytic dataset (N=40879)**	
Variable	%
Father's years of education	0.9
Mother's years of education	0.8
Mother's age (years)	0.0
Father's age (years)	0.0
Maternal depressive/anxiety traits, Hopkins Symptoms Checklist-25 (SCL-25)	3.1
Paternal depressive/anxiety traits, Hopkins Symptoms Checklist-25 (SCL-25)	7.0
Maternal ADHD traits: adult ADHD self-report scale	40.9
Paternal ADHD traits: adult ADHD self-report scale	60.0
Child depressive traits age 8: Short Mood and Feelings Questionnaire (SMFQ)[f]	54.0
Child anxiety traits age 8: Screen for Child Anxiety Related Disorders (SCARED)	54.0
Child ADHD traits age 8: Parent/Teacher Rating Scale for Disruptive Behaviour Disorders (RS-DBD)	54.0
Child ADHD traits (inattention) age 8: Parent/Teacher Rating Scale for Disruptive Behaviour Disorders (RS-DBD)	54.1
Child's sex	0.0
Mother's parity at child's birth	0.0
Mother's marital status at child's birth	0.0
Mother's smoking status during pregnancy	2.0
Father's smoking status during pregnancy	0.7

Supplementary Table 3: Maternal and paternal education (years) and children's traits of depression, anxiety, and ADHD at age 8 in MoBa (N=40,879)[a]

	Non-genetic estimate[b]			Non-genetic estimate, adjusted[c]			MR estimate, adjusted for co-parent's polygenic index[d]			Within-family MR estimate: genetic trio model[e]		
**Depressive traits: standardized Short Mood and Feelings Questionnaire (SMFQ)[d] score.**												
	**Beta**	**CI**	**p**	**Beta**	**CI**	**p**	**Beta**	**CI**	**p**	**Beta**	**CI**	**p**
Mother's education (years)	-0.02	-0.02,-0.01	<0.001	-0.00	-0.01,0.00	0.222	-0.02	-0.06,0.03	0.443	0.00	-0.04,0.05	0.94
Father's education (years)	-0.02	-0.02,-0.01	<0.001	-0.01	-0.02,-0.00	0.002	-0.04	-0.08,0.00	0.050	-0.02	-0.06,0.02	0.386
Child's polygenic index										-0.04	-0.06,-0.02	0.001
**Anxiety traits: standardized Short Screen for Child Anxiety Related Disorders (SCARED)[e] score.**												
	**Beta**	**CI**	**p**	**Beta**	**CI**	**p**	**Beta**	**CI**	**p**	**Beta**	**CI**	**p**
Mother's education (years)	-0.01	-0.02,-0.00	0.008	-0.01	-0.01,0.00	0.102	-0.02	-0.07,0.03	0.486	-0.01	-0.06,0.05	0.752
Father's education (years)	-0.00	-0.01,0.01	0.903	0.00	-0.00,0.01	0.467	0.00	-0.04,0.05	0.905	0.01	-0.03,0.06	0.545
Child's polygenic index										-0.02	-0.04,0.00	0.054
**ADHD traits: standardized Parent/Teacher Rating Scale for Disruptive Behaviour Disorders (RS-DBD)[f] score.**												
	**Beta**	**CI**	**p**	**Beta**	**CI**	**p**	**Beta**	**CI**	**p**	**Beta**	**CI**	**p**
Mother's education (years)	-0.02	-0.03,-0.01	<0.001	-0.01	-0.01,0.00	0.154	-0.01	-0.06,0.04	0.764	0.03	-0.02,0.08	0.223
Father's education (years)	-0.03	-0.03,-0.02	<0.001	-0.02	-0.02,-0.01	<0.001	-0.06	-0.10,-0.02	0.003	-0.02	-0.06,0.02	0.322
Child's polygenic index										-0.08	-0.09,-0.06	<0.001
**ADHD-inattention traits: standardized Parent/Teacher Rating Scale for Disruptive Behaviour Disorders (RS-DBD)[f] score, inattention items.**												
	**Beta**	**CI**	**p**	**Beta**	**CI**	**p**	**Beta**	**CI**	**p**	**Beta**	**CI**	**p**
Mother's education (years)	-0.01	-0.02,-0.01	<0.001	0.00	-0.01,0.01	0.546	0.02	-0.03,0.07	0.483	0.06	0.01,0.11	0.018
Father's education (years)	-0.03	-0.03,-0.02	<0.001	-0.02	-0.02,-0.01	<0.001	-0.08	-0.12,-0.04	<0.001	-0.03	-0.08,0.01	0.116
Child's polygenic index										-0.09	-0.11,-0.07	<0.001
**ADHD-inattention traits: standardized Parent/Teacher Rating Scale for Disruptive Behaviour Disorders (RS-DBD)[f] score, hyperactivity items.**												
	**Beta**	**CI**	**p**				**Beta**	**CI**	**p**	**Beta**	**CI**	**p**
Mother's education (years)	-0.02	-0.03,-0.01	<0.001	-0.01	-0.01,0.00	0.043	-0.03	-0.08,0.02	0.190	-0.01	-0.06,0.04	0.737
Father's education (years)	-0.02	-0.02,-0.01	<0.001	-0.01	-0.02,-0.00	0.001	-0.03	-0.07,0.01	0.161	0.00	-0.05,0.04	0.883
Child's polygenic index										-0.05	-0.07,-0.03	<0.001

[a]Coefficients represent S.D. change in child's traits per one-year increase in parental years of education. [b]Non-genetic estimate: adjusted for the child's sex and birth year, and the child's, mother's, and father's genotyping centre, genotyping chip, and first 20 principal components of ancestry. [c]Non-genetic estimate, adjusted: also adjusted for the mother's and father's depressive/anxiety and ADHD traits and smoking status during pregnancy. [d]MR estimate, adjusted for co-parent's polygenic index: adjusted for the child's sex and birth year, the child's, mother's, and father's genotyping centre, genotyping chip, and first 20 principal components of ancestry, and the co-parent's education polygenic index. [e]Within-family MR: genetic trio model: adjusted for the child's sex and birth year, the child's, mother's, and father's genotyping centre, genotyping chip, and first 20 principal components of ancestry, the co-parent's education polygenic index, and the child's own education polygenic index.

Supplementary Table 4: Maternal and paternal education (years) and female children's traits of depression, anxiety, and ADHD at age 8 in MoBa (N=20,013)[a]

	Non-genetic estimate[b]			Non-genetic estimate, adjusted[c]			MR estimate, adjusted for co-parent's polygenic index[d]			Within-family MR estimate: genetic trio model[e]		
**Depressive traits: standardized Short Mood and Feelings Questionnaire (SMFQ)[d] score.**												
	**Beta**	**CI**	**p**	**Beta**	**CI**	**p**	**Beta**	**CI**	**p**	**Beta**	**CI**	**p**
Mother's education (years)	-0.02	-0.03,-0.01	<0.001	-0.01	-0.02,-0.00	0.033	-0.04	-0.11,0.03	0.250	-0.02	-0.09,0.05	0.567
Father's education (years)	-0.01	-0.02,0.00	0.066	0.00	-0.01,0.01	0.933	-0.02	-0.08,0.04	0.557	0.01	-0.05,0.07	0.824
Child's polygenic index										-0.04	-0.07,-0.01	0.005
**Anxiety traits: standardized Short Screen for Child Anxiety Related Disorders (SCARED)[e] score.**												
	**Beta**	**CI**	**p**	**Beta**	**CI**	**p**	**Beta**	**CI**	**p**	**Beta**	**CI**	**p**
Mother's education (years)	-0.01	-0.02,0.00	0.063	-0.01	-0.02,0.00	0.188	-0.03	-0.11,0.04	0.382	-0.02	-0.09,0.06	0.656
Father's education (years)	0.00	-0.01,0.01	0.984	0.00	-0.01,0.01	0.631	0.02	-0.04,0.08	0.544	0.04	-0.03,0.10	0.248
Child's polygenic index										-0.03	-0.07,-0.00	0.040
**ADHD traits: standardized Parent/Teacher Rating Scale for Disruptive Behaviour Disorders (RS-DBD)[f] score.**												
	**Beta**	**CI**	**p**	**Beta**	**CI**	**p**	**Beta**	**CI**	**p**	**Beta**	**CI**	**p**
Mother's education (years)	-0.03	-0.04,-0.02	<0.001	-0.01	-0.02,-0.00	0.048	-0.01	-0.08,0.06	0.767	0.03	-0.04,0.10	0.444
Father's education (years)	-0.02	-0.03,-0.02	<0.001	-0.01	-0.02,-0.01	0.001	-0.06	-0.12,-0.00	0.034	-0.01	-0.07,0.05	0.663
Child's polygenic index										-0.08	-0.11,-0.05	<0.001
**ADHD-inattention traits: standardized Parent/Teacher Rating Scale for Disruptive Behaviour Disorders (RS-DBD)[f] score, inattention items.**												
	**Beta**	**CI**	**p**	**Beta**	**CI**	**p**	**Beta**	**CI**	**p**	**Beta**	**CI**	**p**
Mother's education (years)	-0.02	-0.03,-0.01	<0.001	-0.01	-0.02,0.00	0.282	0.02	-0.05,0.09	0.499	0.07	-0.01,0.14	0.068
Father's education (years)	-0.03	-0.03,-0.02	<0.001	-0.02	-0.03,-0.01	<0.001	-0.09	-0.15,-0.04	0.001	-0.04	-0.10,0.02	0.168
Child's polygenic index										-0.09	-0.12,-0.06	<0.001
**ADHD-inattention traits: standardized Parent/Teacher Rating Scale for Disruptive Behaviour Disorders (RS-DBD)[f] score, hyperactivity items.**												
	**Beta**	**CI**	**p**				**Beta**	**CI**	**p**	**Beta**	**CI**	**p**
Mother's education (years)	-0.03	-0.04,-0.02	<0.001	-0.01	-0.02,-0.00	0.017	-0.04	-0.12,0.03	0.270	-0.02	-0.10,0.06	0.645
Father's education (years)	-0.02	-0.02,-0.01	<0.001	-0.01	-0.01,0.00	0.146	-0.01	-0.08,0.05	0.669	0.02	-0.05,0.09	0.613
Child's polygenic index										-0.05	-0.08,-0.02	<0.001

[a]Coefficients represent S.D. change in child's traits per one-year increase in parental years of education. [b]Non-genetic estimate: adjusted for the child's sex and birth year, and the child's, mother's, and father's genotyping centre, genotyping chip, and first 20 principal components of ancestry. [c]Non-genetic estimate, adjusted: also adjusted for the mother's and father's depressive/anxiety and ADHD traits and smoking status during pregnancy. [d]MR estimate, adjusted for co-parent's polygenic index: adjusted for the child's sex and birth year, the child's, mother's, and father's genotyping centre, genotyping chip, and first 20 principal components of ancestry, and the co-parent's education polygenic index. [e]Within-family MR: genetic trio model: adjusted for the child's sex and birth year, the child's, mother's, and father's genotyping centre, genotyping chip, and first 20 principal components of ancestry, the co-parent's education polygenic index, and the child's own education polygenic index.

Supplementary Table 5: Maternal and paternal education (years) and male children's traits of depression, anxiety, and ADHD at age 8 in MoBa (N=20,866)[a]

	Non-genetic estimate[b]			Non-genetic estimate, adjusted[c]			MR estimate, adjusted for co-parent's polygenic index[d]			Within-family MR estimate: genetic trio model[e]		
**Depressive traits: standardized Short Mood and Feelings Questionnaire (SMFQ)[d] score.**												
	**Beta**	**CI**	**p**	**Beta**	**CI**	**p**	**Beta**	**CI**	**p**	**Beta**	**CI**	**p**
Mother's education (years)	-0.01	-0.02,0.00	0.123	0.00	-0.01,0.01	0.826	0.01	-0.07,0.08	0.837	0.03	-0.05,0.10	0.485
Father's education (years)	-0.03	-0.04,-0.02	<0.001	-0.02	-0.03,-0.01	<0.001	-0.06	-0.12,-0.00	0.043	-0.04	-0.10,0.02	0.153
Child's polygenic index										-0.03	-0.06,-0.01	0.010
**Anxiety traits: standardized Short Screen for Child Anxiety Related Disorders (SCARED)[e] score.**												
	**Beta**	**CI**	**p**	**Beta**	**CI**	**p**	**Beta**	**CI**	**p**	**Beta**	**CI**	**p**
Mother's education (years)	-0.01	-0.02,0.00	0.122	-0.00	-0.01,0.01	0.439	-0.01	-0.08,0.06	0.816	-0.01	-0.08,0.07	0.859
Father's education (years)	-0.00	-0.01,0.01	0.862	0.00	-0.01,0.01	0.642	-0.01	-0.07,0.05	0.834	-0.01	-0.07,0.06	0.883
Child's polygenic index										-0.01	-0.03,0.03	0.807
**ADHD traits: standardized Parent/Teacher Rating Scale for Disruptive Behaviour Disorders (RS-DBD)[f] score.**												
	**Beta**	**CI**	**p**	**Beta**	**CI**	**p**	**Beta**	**CI**	**p**	**Beta**	**CI**	**p**
Mother's education (years)	-0.01	-0.02,-0.00	0.012	-0.00	-0.01,0.01	0.851	0.01	-0.07,0.08	0.845	0.04	-0.03,0.12	0.250
Father's education (years)	-0.03	-0.03,-0.02	<0.001	-0.02	-0.02,-0.01	<0.001	-0.07	-0.13,-0.01	0.024	-0.04	-0.10,0.03	0.243
Child's polygenic index										-0.07	-0.10,-0.04	<0.001
**ADHD-inattention traits: standardized Parent/Teacher Rating Scale for Disruptive Behaviour Disorders (RS-DBD)[f] score, inattention items.**												
	**Beta**	**CI**	**p**	**Beta**	**CI**	**p**	**Beta**	**CI**	**p**	**Beta**	**CI**	**p**
Mother's education (years)	-0.01	-0.02,0.00	0.055	0.00	-0.01,0.01	0.931	0.02	-0.06,0.10	0.631	0.06	-0.02,0.14	0.118
Father's education (years)	-0.02	-0.03,-0.02	<0.001	-0.02	-0.02,-0.01	<0.001	-0.08	-0.14,-0.01	0.022	-0.03	-0.10,0.03	0.298
Child's polygenic index										-0.08	-0.11,-0.05	<0.001
**ADHD-inattention traits: standardized Parent/Teacher Rating Scale for Disruptive Behaviour Disorders (RS-DBD)[f] score, hyperactivity items.**												
	**Beta**	**CI**	**p**				**Beta**	**CI**	**p**	**Beta**	**CI**	**p**
Mother's education (years)	-0.01	-0.02,-0.00	0.012	-0.00	-0.01,0.01	0.663	-0.01	-0.08,0.06	0.862	0.02	-0.05,0.09	0.647
Father's education (years)	-0.02	-0.03,-0.01	<0.001	-0.01	-0.02,-0.00	0.002	-0.05	-0.11,0.01	0.074	-0.03	-0.09,0.03	0.281
Child's polygenic index										-0.04	-0.07,-0.02	0.001

[a]Coefficients represent S.D. change in child's traits per one-year increase in parental years of education. [b]Non-genetic estimate: adjusted for the child's sex and birth year, and the child's, mother's, and father's genotyping centre, genotyping chip, and first 20 principal components of ancestry. [c]Non-genetic estimate, adjusted: also adjusted for the mother's and father's depressive/anxiety and ADHD traits and smoking status during pregnancy. [d]MR estimate, adjusted for co-parent's polygenic index: adjusted for the child's sex and birth year, the child's, mother's, and father's genotyping centre, genotyping chip, and first 20 principal components of ancestry, and the co-parent's education polygenic index. [e]Within-family MR: genetic trio model: adjusted for the child's sex and birth year, the child's, mother's, and father's genotyping centre, genotyping chip, and first 20 principal components of ancestry, the co-parent's education polygenic index, and the child's own education polygenic index.

Supplementary Table 6: Maternal and paternal education (years) and children's square root-transformed traits of depression, anxiety, and ADHD at age 8 in MoBa (N=40,879)[a]

	Non-genetic estimate[b]			Non-genetic estimate, adjusted[c]			MR estimate, adjusted for co-parent's polygenic index[d]			Within-family MR estimate: genetic trio model[e]		
**Depressive traits: standardized Short Mood and Feelings Questionnaire (SMFQ)[d] score.**												
	**Beta**	**CI**	**p**	**Beta**	**CI**	**p**	**Beta**	**CI**	**p**	**Beta**	**CI**	**p**
Mother's education (years)	-0.01	-0.02,-0.01	<0.001	-0.00	-0.01,0.00	0.462	-0.00	-0.05,0.04	0.883	0.02	-0.03,0.06	0.486
Father's education (years)	-0.02	-0.02,-0.01	<0.001	-0.01	-0.01,-0.00	0.001	-0.04	-0.08,-0.01	0.017	-0.02	-0.06,0.01	0.216
Child's polygenic index										-0.04	-0.06,-0.02	0.001
**Anxiety traits: standardized Short Screen for Child Anxiety Related Disorders (SCARED)[e] score.**												
	**Beta**	**CI**	**p**	**Beta**	**CI**	**p**	**Beta**	**CI**	**p**	**Beta**	**CI**	**p**
Mother's education (years)	-0.01	-0.01,-0.00	0.016	-0.00	-0.01,0.00	0.160	-0.01	-0.05,0.02	0.541	-0.00	-0.04,0.03	0.789
Father's education (years)	0.00	-0.00,0.00	0.756	0.00	-0.00,0.01	0.302	0.00	-0.02,0.03	0.758	0.01	-0.02,0.04	0.462
Child's polygenic index										-0.01	-0.03,0.00	0.098
**ADHD traits: standardized Parent/Teacher Rating Scale for Disruptive Behaviour Disorders (RS-DBD)[f] score.**												
	**Beta**	**CI**	**p**	**Beta**	**CI**	**p**	**Beta**	**CI**	**p**	**Beta**	**CI**	**p**
Mother's education (years)	-0.02	-0.03,-0.01	<0.001	-0.00	-0.01,0.00	0.281	0.01	-0.06,0.07	0.841	0.06	-0.01,0.12	0.074
Father's education (years)	-0.03	-0.04,-0.02	<0.001	-0.02	-0.03,-0.01	<0.001	-0.09	-0.09,-0.14	<0.001	-0.04	-0.09,0.01	0.162
Child's polygenic index										-0.10	-0.12,-0.08	<0.001
**ADHD-inattention traits: standardized Parent/Teacher Rating Scale for Disruptive Behaviour Disorders (RS-DBD)[f] score, inattention items.**												
	**Beta**	**CI**	**p**	**Beta**	**CI**	**p**	**Beta**	**CI**	**p**	**Beta**	**CI**	**p**
Mother's education (years)	-0.01	-0.02,-0.00	0.002	-0.00	-0.01,0.01	0.884	0.03	-0.02,0.08	0.242	0.08	0.02,0.13	0.004
Father's education (years)	-0.02	-0.03,-0.02	<0.001	-0.02	-0.02,-0.01	<0.001	-0.09	-0.13,-0.05	<0.001	-0.04	-0.08,-0.00	0.049
Child's polygenic index										-0.09	-0.11,-0.07	<0.001
**ADHD-inattention traits: standardized Parent/Teacher Rating Scale for Disruptive Behaviour Disorders (RS-DBD)[f] score, hyperactivity items.**												
	**Beta**	**CI**	**p**				**Beta**	**CI**	**p**	**Beta**	**CI**	**p**
Mother's education (years)	-0.02	-0.03,-0.01	<0.001	-0.01	-0.01,0.00	0.060	-0.03	-0.08,0.03	0.306	-0.00	-0.06,0.05	0.935
Father's education (years)	-0.02	-0.03,-0.01	<0.001	-0.01	-0.02,-0.00	0.004	-0.04	-0.08,0.01	0.110	-0.01	-0.05,0.04	0.730
Child's polygenic index										-0.05	-0.07,-0.03	<0.001

[a]Coefficients represent change in child's square root-transformed traits per one-year increase in parental years of education. [b]Non-genetic estimate: adjusted for the child's sex and birth year, and the child's, mother's, and father's genotyping centre, genotyping chip, and first 20 principal components of ancestry. [c]Non-genetic estimate, adjusted: also adjusted for the mother's and father's depressive/anxiety and ADHD traits and smoking status during pregnancy. [d]MR estimate, adjusted for co-parent's polygenic index: adjusted for the child's sex and birth year, the child's, mother's, and father's genotyping centre, genotyping chip, and first 20 principal components of ancestry, and the co-parent's education polygenic index. [e]Within-family MR: genetic trio model: adjusted for the child's sex and birth year, the child's, mother's, and father's genotyping centre, genotyping chip, and first 20 principal components of ancestry, the co-parent's education polygenic index, and the child's own education polygenic index.

Supplementary Table 7: Maternal and paternal education (years) and children's traits of depression, anxiety, and ADHD at age 8 in MoBa, complete-case analysis[a]

	Non-genetic estimate[b]			Non-genetic estimate, adjusted[c]			MR estimate, adjusted for co-parent's polygenic index[d]			Within-family MR estimate: genetic trio model[e]		
**Depressive traits: standardized Short Mood and Feelings Questionnaire (SMFQ)[d] score. N=6,295**												
	**Beta**	**CI**	**p**	**Beta**	**CI**	**p**	**Beta**	**CI**	**p**	**Beta**	**CI**	**p**
Mother's education (years)	-0.01	-0.02,0.00	0.124	-0.00	-0.02,0.01	0.593	0.14	0.00,0.28	0.055	0.16	0.02,0.30	0.025
Father's education (years)	-0.01	-0.02,0.00	0.088	-0.00	-0.01,0.01	0.492	-0.10	-0.20,-0.01	0.033	-0.09	-0.19,0.01	0.080
Child's polygenic index										-0.03	-0.07,0.01	0.106
**Anxiety traits: standardized Short Screen for Child Anxiety Related Disorders (SCARED)[e] score. N=6311**												
	**Beta**	**CI**	**p**	**Beta**	**CI**	**p**	**Beta**	**CI**	**p**	**Beta**	**CI**	**p**
Mother's education (years)	-0.01	-0.02,0.01	0.241	-0.01	-0.02,0.01	0.436	0.05	-0.09,0.18	0.517	0.05	-0.09,0.19	0.515
Father's education (years)	0.01	0.00,0.02	0.096	0.01	0.00,0.02	0.089	-0.02	-0.11,0.08	0.711	-0.02	-0.12,0.08	0.726
Child's polygenic index										0	-0.04,0.04	0.957
**ADHD traits: standardized Parent/Teacher Rating Scale for Disruptive Behaviour Disorders (RS-DBD)[f] score, ADHD items. N=6308**												
	**Beta**	**CI**	**p**	**Beta**	**CI**	**p**	**Beta**	**CI**	**p**	**Beta**	**CI**	**p**
Mother's education (years)	-0.01	-0.02,0.01	0.236	0.00	-0.01,0.01	0.953	0.07	-0.07,0.21	0.321	0.12	-0.02,0.26	0.092
Father's education (years)	-0.02	-0.03,-0.01	<0.001	-0.01	-0.03,-0.00	0.009	-0.08	-0.17,0.01	0.098	-0.05	-0.14,0.05	0.340
Child's polygenic index										-0.06	-0.10,-0.03	0.001
**ADHD-inattention traits: standardized Parent/Teacher Rating Scale for Disruptive Behaviour Disorders (RS-DBD)[f] score, inattention items. N=6302**												
	**Beta**	**CI**	**p**	**Beta**	**CI**	**p**	**Beta**	**CI**	**p**	**Beta**	**CI**	**p**
Mother's education (years)	-0.01	-0.02,0.01	0.269	0.00	-0.01,0.01	0.934	0.14	0.00,0.28	0.054	0.21	0.06,0.35	0.006
Father's education (years)	-0.02	-0.03,-0.01	0.002	-0.01	-0.02,-0.00	0.027	-0.12	-0.21,-0.02	0.018	-0.08	-0.18,0.03	0.145
Child's polygenic index										-0.08	-0.12,-0.05	<0.001
**ADHD-inattention traits: standardized Parent/Teacher Rating Scale for Disruptive Behaviour Disorders (RS-DBD)[f] score, hyperactivity items. N=6302**												
	**Beta**	**CI**	**p**	**Beta**	**CI**	**p**	**Beta**	**CI**	**p**	**Beta**	**CI**	**p**
Mother's education (years)	-0.01	-0.02,0.01	0.326	0.00	-0.01,0.01	0.985	-0.02	-0.16,0.12	0.769	0.00	-0.13,0.14	0.972
Father's education (years)	-0.02	-0.03,-0.01	0.001	-0.01	-0.03,-0.00	0.014	-0.02	-0.11,0.07	0.648	-0.01	-0.10,0.09	0.877
Child's polygenic index										-0.03	-0.07,0.01	0.139

[a]Coefficients represent S.D. change in child's traits per one-year increase in parental years of education. [b]Non-genetic estimate: adjusted for the child's sex and birth year, and the child's, mother's, and father's genotyping centre, genotyping chip, and first 20 principal components of ancestry. [c]Non-genetic estimate, adjusted: also adjusted for the mother's and father's depressive/anxiety and ADHD traits and smoking status during pregnancy. [d]MR estimate, adjusted for co-parent's polygenic index: adjusted for the child's sex and birth year, the child's, mother's, and father's genotyping centre, genotyping chip, and first 20 principal components of ancestry, and the co-parent's education polygenic index. [e]Within-family MR: genetic trio model: adjusted for the child's sex and birth year, the child's, mother's, and father's genotyping centre, genotyping chip, and first 20 principal components of ancestry, the co-parent's education polygenic index, and the child's own education polygenic index.

Supplementary Table 8: Results of two-sample summary data Mendelian randomization[a]: 1729 SNPs in educational attainment polygenic index

	**Inverse-variance weighted**		**MR-Egger: slope**		**MR-Egger: intercept**		**MR-Median**		**MR-Modal**	
Maternal education (years)	Beta	CI	Beta	CI	Beta	CI	Beta	CI	Beta	CI
Depressive traits: standardized SMFQ[b] score	-0.04	-0.08,-0.01	-0.09	-0.28,0.11	0.00	0.00,0.00	-0.05	-0.14,0.04	-0.15	-0.47,0.18
Anxiety traits: standardized SCARED[c] score	0.00	-0.03,0.04	-0.05	-0.25,0.15	0.00	0.00,0.00	0.01	-0.08,0.09	-0.07	-0.40,0.26
ADHD traits: standardized RS-DBD[d] score	0.01	-0.03,0.05	0.03	-0.16,0.22	0.00	0.00,0.00	0.00	-0.08,0.09	-0.07	-0.40,0.26
ADHD traits (inattention): standardized RS-DBD[d] score	0.06	0.02,0.10	0.06	-0.14,0.25	0.00	0.00,0.00	0.07	-0.01,0.16	0.15	-0.16,0.47
ADHD traits (hyperactivity): standardized RS-DBD[d] score	-0.04	-0.08,0.00	-0.01	-0.21,0.18	0.00	0.00,0.00	-0.04	-0.13,0.04	-0.07	-0.40,0.25
	**Inverse-variance weighted**		**MR-Egger: slope**		**MR-Egger: intercept**		**MR-Median**		**MR-Modal**	
Paternal education (years)	Beta	CI	Beta	CI	Beta	CI	Beta	CI	Beta	CI
Depressive traits: standardized SMFQ[b] score	-0.06	-0.10,-0.03	-0.05	-0.25,0.14	0.00	0.00,0.00	-0.08	-0.17,0.00	-0.15	-0.48,0.18
Anxiety traits: standardized SCARED[c] score	0.02	-0.02,0.05	0.10	-0.10,0.29	0.00	0.00,0.00	0.03	-0.06,0.11	0.04	-0.29,0.36
ADHD traits: standardized RS-DBD[d] score	-0.04	-0.07,0.00	-0.07	-0.26,0.12	0.00	0.00,0.00	-0.05	-0.13,0.04	-0.03	-0.36,0.30
ADHD traits (inattention): standardized RS-DBD[d] score	-0.03	-0.07,0.01	-0.05	-0.24,0.14	0.00	0.00,0.00	-0.07	-0.15,0.02	-0.17	-0.50,0.16
ADHD traits (hyperactivity): standardized RS-DBD[d] score	-0.04	-0.07,0.00	-0.07	-0.26,0.12	0.00	0.00,0.00	-0.05	-0.13,0.04	0.01	-0.31,0.33

[a]Coefficients represent S.D. change in child's traits per one-year increase in parental years of schooling. [b]Short Mood and Feelings Questionnaire. [c]Screen for Child Anxiety Related Disorders. [d]Parent/Teacher Rating Scale for Disruptive Behaviour Disorders.

Supplementary Table 9: Results of two-sample summary data Mendelian randomization[a]: 510 SNPs in educational attainment polygenic index[b]

	**Inverse-variance weighted**		**MR-Egger: slope**		**MR-Egger: intercept**		**MR-Median**		**MR-Modal**	
Maternal education (years)	Beta	CI	Beta	CI	Beta	CI	Beta	CI	Beta	CI
Depressive traits: standardized SMFQ[c] score	-0.02	-0.07,0.04	-0.17	-0.46,0.12	0.00	0.00,0.01	-0.04	-0.18,0.09	-0.12	-0.49,0.25
Anxiety traits: standardized SCARED[d] score	0.01	-0.05,0.06	-0.05	-0.34,0.25	0.00	0.00,0.00	0.03	-0.10,0.16	0.07	-0.29,0.44
ADHD traits: standardized RS-DBD[e] score	0.04	-0.02,0.10	0.05	-0.24,0.33	0.00	0.00,0.00	0.01	-0.12,0.14	-0.10	-0.46,0.27
ADHD traits (inattention): standardized RS-DBD[e] score	0.07	0.02,0.13	0.12	-0.17,0.40	0.00	0.00,0.00	0.10	-0.03,0.23	0.13	-0.20,0.46
ADHD traits (hyperactivity): standardized RS-DBD[e] score	0.00	-0.06,0.06	-0.05	-0.33,0.24	0.00	0.00,0.00	-0.04	-0.17,0.09	-0.13	-0.49,0.23
	**Inverse-variance weighted**		**MR-Egger: slope**		**MR-Egger: intercept**		**MR-Median**		**MR-Modal**	
Paternal education (years)	Beta	CI	Beta	CI	Beta	CI	Beta	CI	Beta	CI
Depressive traits: standardized SMFQ[c] score	-0.04	-0.10,0.01	0.04	-0.25,0.33	0.00	0.00,0.00	-0.05	-0.18,0.08	-0.15	-0.52,0.22
Anxiety traits: standardized SCARED[d] score	0.05	-0.01,0.10	0.14	-0.15,0.43	0.00	0.00,0.00	0.05	-0.08,0.18	0.05	-0.29,0.40
ADHD traits: standardized RS-DBD[e] score	-0.03	-0.09,0.02	-0.13	-0.41,0.15	0.00	0.00,0.00	-0.05	-0.18,0.08	-0.04	-0.40,0.32
ADHD traits (inattention): standardized RS-DBD[e] score	-0.03	-0.08,0.03	-0.05	-0.33,0.24	0.00	0.00,0.00	-0.07	-0.19,0.06	-0.16	-0.53,0.21
ADHD traits (hyperactivity): standardized RS-DBD[e] score	-0.03	-0.09,0.02	-0.19	-0.47,0.10	0.00	0.00,0.00	-0.06	-0.19,0.08	-0.05	-0.42,0.32

[a]Coefficients represent S.D. change in child's traits per one-year increase in parental years of schooling. [b]Subset of SNPs clumped at a more stringent linkage disequilibrium threshold of r^2=0.001. [c]Short Mood and Feelings Questionnaire. [d]Screen for Child Anxiety Related Disorders. [e]Parent/Teacher Rating Scale for Disruptive Behaviour Disorders.

Supplementary notes

Supplementary Note 1: Comparison of included and excluded participants

Compared to those excluded from the sample, mothers and fathers in the analytic sample had on average slightly more years of schooling (by 0.40 years and 0.26 years, both p<0.001). They scored slightly lower for depressive/anxiety traits (maternal Hopkins score: 1.19 vs 1.36, paternal Hopkins score: 1.12 vs 1.23), and mothers scored lower for ADHD traits (6.49 vs 6.63). Retained fathers were slightly younger (32.6, compared to 32.8 years). Mothers did not differ in age but were less likely to have been single at the child's birth (2.6% vs 5.6%), and had had fewer previous pregnancies (e.g., 46.8% vs 42.4% nulliparous). Children in the analytic sample did not differ on sex but tended to be born slightly later (difference: 0.8 years), and had slightly lower traits of depression, anxiety, and ADHD (SMFQ: 1.81 vs 1.91, SCARED: 1.01 vs 1.04, RS-DBD ADHD: 8.36 vs 8.66, all p<0.001).
