# Supplementary material for: Parental education and children’s depression, anxiety, and ADHD traits, a within-family study in MoBa
Source: NPJ Sci Learn. 2024 Jul 18;9:46. doi: 10.1038/s41539-024-00260-8 (PMC11258307; doi:10.1038/s41539-024-00260-8)
Supplement: Supplementary file 2 — Reporting Summary [file 41539_2024_260_MOESM2_ESM.pdf]

Corresponding author(s): Amanda M Hughes
Last updated by author(s): Jun 21, 2024

Reporting Summary

Nature Portfolio wishes to improve the reproducibility of the work that we publish. This form provides structure for consistency and transparency in reporting. For further information on Nature Portfolio policies, see our Editorial Policies and the Editorial Policy Checklist.

Statistics

For all statistical analyses, confirm that the following items are present in the figure legend, table legend, main text, or Methods section.

n/a	Confirmed	
☐	☒	The exact sample size (*n*) for each experimental group/condition, given as a discrete number and unit of measurement
☐	☒	A statement on whether measurements were taken from distinct samples or whether the same sample was measured repeatedly
☒	☐	The statistical test(s) used AND whether they are one- or two-sided<br>*Only common tests should be described solely by name; describe more complex techniques in the Methods section.*
☐	☒	A description of all covariates tested
☒	☐	A description of any assumptions or corrections, such as tests of normality and adjustment for multiple comparisons
☐	☒	A full description of the statistical parameters including central tendency (e.g. means) or other basic estimates (e.g. regression coefficient) AND variation (e.g. standard deviation) or associated estimates of uncertainty (e.g. confidence intervals)
☐	☒	For null hypothesis testing, the test statistic (e.g. F, t, r) with confidence intervals, effect sizes, degrees of freedom and P value noted<br>*Give P values as exact values whenever suitable.*
☒	☐	For Bayesian analysis, information on the choice of priors and Markov chain Monte Carlo settings
☒	☐	For hierarchical and complex designs, identification of the appropriate level for tests and full reporting of outcomes
☒	☐	Estimates of effect sizes (e.g. Cohen's d, Pearson's r), indicating how they were calculated

Our web collection on statistics for biologists contains articles on many of the points above.

Software and code

Policy information about availability of computer code

Data collection	This study was entirely based on existing data, therefore no code was needed for data collection
Data analysis	Analysis was conducted in Stata

For manuscripts utilizing custom algorithms or software that are central to the research but not yet described in published literature, software must be made available to editors and reviewers. We strongly encourage code deposition in a community repository (e.g. GitHub). See the Nature Portfolio guidelines for submitting code & software for further information.

Data

Policy information about availability of data

All manuscripts must include a data availability statement. This statement should provide the following information, where applicable:

- Accession codes, unique identifiers, or web links for publicly available datasets
- A description of any restrictions on data availability
- For clinical datasets or third party data, please ensure that the statement adheres to our policy

MoBa data is not publicly available because the consent given by participants does not allow for data storage on an individual level in repositories or journals. Researchers who want access to the data sets for replication should apply to datailgang@fhi.no. Access to data sets requires approval from The Regional Committee for Medical and Health Research Ethics in Norway and an agreement with MoBa.

Research involving human participants, their data, or biological material

Policy information about studies with human participants or human data. See also policy information about sex, gender (identity/presentation), and sexual orientation and race, ethnicity and racism.

Reporting on sex and gender	Biological sex (derived from chromosomal data) was included as a covariate in all models; we therefore refer to groups of female and male children (not girls and boys). In additional analyses we also stratified on this to examine sex-specific associations.
Reporting on race, ethnicity, or other socially relevant groupings	Neither race nor ethnicity were included in analysis as a covariate. As with most genetic analysis of this sort, the MoBa genetic data is restricted to people of european ancestry to reduce confounding. Genetic principal components capturing finer-grained ancestry were included in models.
Population characteristics	See above
Recruitment	Pregnant women from all over Norway were recruited in 1998-2008. As discussed in the paper, participation was not random: Previous comparison of the MoBa cohort with all women giving birth in Norway has shown that MoBa participants differed from the general population on demographic and health-related factors: mothers who were younger or living alone were underrepresented, as were smokers, and women who had had more previous pregnancies. Women who used multivitamins were overrepresented.
Ethics oversight	The establishment of MoBa and initial data collection was based on a license from the Norwegian Data Protection Agency and approval from The Regional Committees for Medical and Health Research Ethics. The MoBa cohort is currently regulated by the Norwegian Health Registry Act. The current study was approved by The Regional Committees for Medical and Health Research Ethics (2016/1702).

Note that full information on the approval of the study protocol must also be provided in the manuscript.

Field-specific reporting

Please select the one below that is the best fit for your research. If you are not sure, read the appropriate sections before making your selection.

☐ Life sciences ☒ Behavioural & social sciences ☐ Ecological, evolutionary & environmental sciences

For a reference copy of the document with all sections, see nature.com/documents/nr-reporting-summary-flat.pdf

Behavioural & social sciences study design

All studies must disclose on these points even when the disclosure is negative.

Study description	Quantitative: multivariable regression and genetic instrumental variable analysis.
Research sample	Pregnant women recruited from all over Norway in 1998-2008, their children and their children's fathers.
Sampling strategy	This study was entirely based on existing data: the largest existing study (to out knowledge) with this kind of data. For this reason, no sample size calculations relevant to data collection or sampling were conducted as part of this study.
Data collection	Most information was reported by questionnaires completed by the mother or the father of the child. Linked information from birth records and educational administrative data was also used. Genetic data was obtained from blood samples were obtained from both parents during pregnancy and from mothers and children (umbilical cord) at birth.
Timing	Recruitment occurred in 1998-2008. The first child was born in October 1999 and the last in July 2009. The current study is based on version 12 of the quality-assured data files released for research in January 2019. This analysis used data collected up until when the children were aged 8 years.
Data exclusions	exclusions were made if 1) no questionnaires were completed, or 2) complete genetic data was not available for the mother-father-child trio needed for within-family analysis
Non-participation	At baseline, around 41% of eligible women were recruited into the study, the rest chose not to participate.
Randomization	This was an observational study, so no randomization was performed.

Reporting for specific materials, systems and methods

We require information from authors about some types of materials, experimental systems and methods used in many studies. Here, indicate whether each material, system or method listed is relevant to your study. If you are not sure if a list item applies to your research, read the appropriate section before selecting a response.

Materials & experimental systems

n/a	Involved in the study
☒	☐ Antibodies
☒	☐ Eukaryotic cell lines
☒	☐ Palaeontology and archaeology
☒	☐ Animals and other organisms
☒	☐ Clinical data
☒	☐ Dual use research of concern
☒	☐ Plants

Methods

n/a	Involved in the study
☒	☐ ChIP-seq
☒	☐ Flow cytometry
☒	☐ MRI-based neuroimaging

Plants

Seed stocks	This study did not use plants
Novel plant genotypes	This study did not use plants
Authentication	This study did not use plants
